# A multi-omics analysis reveals CLSPN is associated with prognosis, immune microenvironment and drug resistance in cancers

**DOI:** 10.1186/s12575-023-00201-6

**Published:** 2023-06-03

**Authors:** Yihong Chen, Haicheng Wen, Yin Li, Ying Han, Jun Tan, Cao Guo, Changjing Cai, Ping Liu, Yinghui Peng, Yihan Liu, Xinwen Wang, Shan Zeng, Ziyang Feng, Hong Shen

**Affiliations:** 1 Department of Oncology, Xiangya Hospital, Central South University, Changsha, 410008 Hunan China; 2 Department of Spine Surgery and Orthopaedics, Xiangya Hospital, Central South University, Changsha, 410008 Hunan China; 3 Department of Neurosurgery, Xiangya Hospital, Central South University, Changsha, 410008 Hunan China; 4 National Clinical Research Center for Geriatric Disorders, Xiangya Hospital, Central South University, Changsha, 410008 Hunan China

**Keywords:** CLSPN, Prognosis, Immune infiltration, Multi-omics, Pan-cancer, Virtual screening

## Abstract

**Background:**

Immunotherapy is effective only in limited patients. It is urgent to discover a novel biomarker to predict immune cells infiltration status and immunotherapy response of different cancers. CLSPN has been reported to play a pivotal role in various biological processes. However, a comprehensive analysis of CLSPN in cancers has not been conducted.

**Methods:**

To show the whole picture of CLSPN in cancers, a pan-cancer analysis was conducted in 9125 tumor samples across 33 cancer types by integrating transcriptomic, epigenomic and pharmacogenomics data. Moreover, the role of CLSPN in cancer was validated by CCK-8, EDU, colony formation and flow cytometry in vitro and tumor cell derived xenograft model in vivo.

**Results:**

CLSPN expression was generally upregulated in most cancer types and was significantly associated with prognosis in different tumor samples. Moreover, elevated CLSPN expression was closely correlated with immune cells infiltration, TMB (tumor mutational burden), MSI (microsatellite instability), MMR (mismatch repair), DNA methylation and stemness score across 33 cancer types. Enrichment analysis of functional genes revealed that CLSPN participated in the regulation of numerous signaling pathways involved in cell cycle and inflammatory response. The expression of CLSPN in LUAD patients were further analyzed at the single-cell level. Knockdown CLSPN significantly inhibited cancer cell proliferation and cell cycle related cyclin-dependent kinase (CDK) family and Cyclin family expression in LUAD (lung adenocarcinoma) both in vitro and in vivo experiments. Finally, we conducted structure-based virtual screening by modelling the structure of CHK1 kinase domain and Claspin phosphopeptide complex. The top five hit compounds were screened and validated by molecular docking and Connectivity Map (CMap) analysis.

**Conclusion:**

Our multi-omics analysis offers a systematic understanding of the roles of CLSPN in pan-cancer and provides a potential target for future cancer treatment.

**Supplementary Information:**

The online version contains supplementary material available at 10.1186/s12575-023-00201-6.

## Introduction

Cancer being a leading cause of morbidity, has become an important threatening factor for worldwide public health. In recent decades, although great progress has been made in the diagnosis and treatment of cancers, the survival rate of patients is still not satisfactory [1]. With the extensive application of cancer genomics databases, it is probable to find more novel tumor biomarkers correlating with clinical prognosis via conducting pan-cancer analysis of genes.

Claspin is originally extracted from Xenopus egg as an important nuclear protein that activates the ATR-CHK1 checkpoint and has been shown to participate in multiple significant biological processes [2, 3]. As a ring-shaped protein, Claspin contains a C-terminal CHK1 and an N-terminal binding domain, which has a high affinity for branched DNA structures [4]. Although CLSPN plays an important role in ensuring accurate genomic replication, maintaining the normal replication rate, promoting the initiation and termination of DNA damage repair, recent studies have shown that CLSPN alteration may lead to genomic instability thus accelerating cancer development [5–7]. Growing researches suggest that CLSPN expression increases in several human cancers and is closely correlated with patients' survival rate [8–10]. Due to the fact that the majority of researches on CLSPN are restricted to particular tumor types, a comprehensive multi-omics investigation of CLSPN in pan-cancer is crucial.

Here, we first performed an integrative pan-cancer analysis to evaluate the diagnosis and prognostic value of CLSPN in various cancers. Furthermore, CLSPN expression was found to correlate with immune cell infiltration levels, tumor mutational burden (TMB), microsatellite instability (MSI), mismatch repair (MMR) gene, DNA methylation and stemness score across 33 types of cancer. We also validated our bioinformatics results through in vitro and in vivo experiments. Last, the FDA-approved drugs targeting Claspin protein complex were investigated by molecular docking analysis and were further validated by Connectivity Map (CMap) analysis. In general, our study systematically analyzed the functions of CLSPN, which might provide potential therapeutic strategies for cancers.

## Methods

### Gene expression analysis

TIMER2 (tumor immune estimation resource, version 2, http://timer.cistrome.org/) and GEPIA2 (Gene Expression Profiling Interactive Analysis, version 2) tool (http://gepia2.cancer-pku.cn/#analysis) was exploited to investigate CLSPN expression profiling spectrum [11, 12]. In addition, CCLE (Cancer Cell Line Encyclopedia, https://portals.broadinstitute.org/ccle/) was used to validate CLSPN in human cancer cell lines. The log2 (transcripts per million (TPM) + 1) transformed expression data were exploited for the box plots [13].

### Survival analysis

Univariate Cox analysis was conducted to investigate the correlation between CLSPN expression and overall survival (OS), disease-specific survival (DSS), disease-free interval (DFI) and progression-free interval (PFI) in all TCGA cancers. The Kaplan–Meier (KM) survival analysis was performed using the R-package “survminer” and “survival” to evaluate the OS, DSS, DFI, PFI for patients with high-CLSPN and low-CLSPN expression, and a *P* < 0.05 indicated statistical significance [14].

### Genetic alteration analysis

The cBioPortal website (https://www.cbioportal.org/) was utilized to investigate CLSPN variation characteristics [15]. Alteration frequency, mutation type, and copy number alteration (CNA) results of all TCGA tumors were obtained from the “cancer types summary” module. The “comparison” module was used to obtain the data on the OS, DFS, PFS, and DSS in all the TCGA cancer types with or without CLSPN alteration.

### Immune infiltration analysis

The ESTIMATE algorithm was exploited to infer the infiltration degree of stromal or immune cells into tumors for each tumor sample, whose results were displayed in the form of Immune score, Stromal score, and Estimate score using the “estimate” and “limma” R packages. In addition, the immune infiltration among different cancer types was obtained by XCELL and CIBERSORT [16, 17].

The immune checkpoint markers level was further acquired for correlation analysis. The “UCSCxenashiny” was used to evaluate TMB and MSI scores [18]. The association between CLSPN expression and TMB or MSI was analyzed by applying Spearman’s method.

### Correlation analysis of CLSPN with DNA mismatch repair (MMR) genes and methylation

Five MMR genes, including MutL homologous gene 1 (MLH1), MutS homologous gene 2 (MSH2), MutS homologous gene 6 (MSH6), postmeiotic segregation increased 2 (PMS2), epithelial cell adhesion molecule (EPCAM), and four methyltransferases (DNMT1, DNMT2, DNMT3A, and DNMT3B) were assessed in different cancers by Spearman’s correlation analysis. Correlation of CLSPN methylation with OS in all TCGA tumor types was conducted using Kaplan–Meier survival analysis (*P* < 0.05 as significant). Besides, MethSurv (https://biit.cs.ut.ee/methsurv/) was used to explore the influence of single CpG methylation of CLSPN gene on the prognosis of LUAD patients [19]. The clustering analysis of individual CpG site of CLSPN in LUAD samples was presented by heatmap using “Gene visualization” tab [20].

### Gene set enrichment analysis (GSEA)

The data from cbioportal online (http://www.cbioportal.org/) was used to conduct functional analysis [21, 22]. Enriched pathways were visualized with R packages “fgsea” and “ggplot2” [23].

### Tumor Immune Single Cell Hub database

Tumor Immune Single Cell Hub (TISCH, http://tisch.comp-genomics.org/) collected 190 tumor single cell datasets and 6297320 cells from GEO and ArrayExpress [24]. In this study, we used the datasets derived from TISCH to comprehensively explore the function of CLSPN on TME heterogeneity in LUAD at single cell level.

### Specimen collection

LUAD tissues and adjacent normal lung tissues were collected via surgical resection from 28 LUAD patients in Xiangya Hospital of Central South University. All the patients were diagnosed by histopathology. This study was approved by Ethics Committees of Xiangya Hospital. The detailed clinicopathological characteristics were described in Table S1.

### Multiplex immunofluorescence (IF) staining

The 4 μm paraffin-embedded lung adenocarcinoma tissue sections were blocked with 3% H2O2 and 3% BSA after undergoing dewaxing, hydration and antigen retrieval. Multiplexed immunofluorescence staining of Claspin (Rabbit, 1:200, abcam, UK), CD8 (Rabbit, 1:400, CST, USA), PD-1 (Rabbit, 1:200, abcam, UK) and PD-L1 (Rabbit, 1:100, abcam, UK) were performed according to the manufacturer's instructions (AiFang biological, 6-Color Multiple fluorescence Kit, China). The images were captured using AKOYA multispectral microscope.

### Cell culture

The human normal pulmonary epithelial cell Beas2B and lung cancer cell lines H1299, Calu-3, SPCA1, HCC827, PC9 and A549 were acquired from the Institutes of Biomedical Sciences (Shanghai, China). Cells were cultured in DMEM or RPMI 1640 added with 10% fetal bovine serum (FBS).

### Plasmid transfection

Transient plasmid transfection was conducted in accordance with the Lipofectamine 3000 reagent instruction (Invitrogen, USA). shRNA targeting CLSPN (sh-CLSPN) and its negative control shRNA (NC) were constructed by GeneChem. After cultured for 48 h, cells mRNA and protein were extracted to validate transfection efficiency.

### Lentivirus infection

The lentiviral vector GV112 (hU6-MCS-CMV-Puromycin) containing human CLSPN-RNAi sequence (NM_001190481) and empty vector were constructed by GeneChem (Shanghai, China). Cell transfection was conducted according to the operating manual. To screen stably transduced cells, the infected cells were incubated in puromycin (2 ug/mL) for 2 weeks. The transfection efficiency of CLSPN-RNAi lentivirus was determined by RT-qPCR.

### RNA isolation and quantitative reverse transcription PCR (RT-qPCR)

Total RNA was extracted with Trizol reagent (AG RNAex Pro Reagent, China), and then reverse transcribed into cDNA in accordance with instructions. RT-qPCR was conducted by SYBR Green Mix (SYBR Green *Pro Taq* HS Premix, China). The primers sequence were presented: CLSPN: F: TGGAGAGTGGGGTCCATTCAT; R: CCGGGGTTTACGTTTGAAGAAA. CCNA2: F: GGTACTGAAGTCCGGGAACC; R: TGCTTTCCAAGGAGGAACGG. CCNB1: F: GCACTTCCTTCGGAGAGCAT; R: TTCTTAGCCAGGTGCTGCAT. CCNB2: F: GCGTGCCATCCTAGTGGATT; R: AGCTTCTTCCGGGAAACTGG. CCNE2: F: TCACTGATGGTGCTTGCAGT; R: GTAAAATGGCACAAGGCAGCA. CDK1: F: CTGGGGTCAGCTCGTTACTC; R: TCCACTTCTGGCCACACTTC. CDK2: F: GCTTTTGGAGTCCCTGTTCG; R: GCGAGTCACCATCTCAGCAA. CDK4: F: TGAAATTGGTGTCGGTGCCT; R: ACCTTGATCTCCCGGTCAGT. CDK6: F: ACAGAGCACCCGAAGTCTTG; R: CTGGGAGTCCAATCACGTCC. ACTIN: F: CCTGGCACCCAGCACAAT; R: GGGCCGGACTCGTCATAC.

### Western blot

Total protein was extracted in RIPA lysis buffer containing phosphatase and protease inhibitor cocktail (NCM Biotech, Shanghai, China). After electrophoresis, the denatured protein was transferred to 0.4 µm Polyvinylidene fluoride (PVDF) membranes under 300 mA for 90 min. Subsequently, the membrane was incubated with primary antibodies and secondary antibody. Finally, the protein signal was visualized utilizing the ChemiDocXRS + System.

### Cell proliferation assay

10μL CCK-8 was incubated with 4.0 × 10^3^cells per 96-well plate for 2 h, and then the cell viability was detected as absorbance at 450 nm in 0 h, 24 h, 48 h, 72 h, 96 h respectively.

EdU assays were conducted by the Cell-light EdU Kit (RiboBio, Guangzhou, China) on the basis of manufacturer instructions. After incubating with 50 μM EdU at 37℃ for 4 h, 2 × 10^4^ cells were stained with Apollo dye solution for 30 min. EdU-positive cells were observed by DMi8 microscope.

### Colony formation assay

1.0 × 10^3^ lung adenocarcinoma cells were seeded in a 6‐well plate and cultured at 37 °C for 14 days. Then, the cell colonies were fixed with 4% paraformaldehyde for 15 min and stained with crystal violet solution for 30 min. The numbers of colonies (≥ 50 cells) were calculated by ImageJ software. The assay was executed in triplicate.

### Cell cycle analysis

The collected cells were dealt with 1 ml of DNA Staining solution and 10 μl of Permeabilization solution (MULTI SCIENCES, Zhejiang, China) at room temperature for 30 min. Subsequently, the cell cycle analysis was conducted by the flow cytometer.

### Immunohistochemistry

Xenograft tumor tissue sections were dewaxed and hydrated. After antigen retrieval, the slices were treated with endogenous peroxidase blocker for 20 min and then incubated with primary antibodies at 4 °C overnight by the following antibodies: Claspin (1:400, abcam), CCNA2 (1:400, abcam), CCNB1 (1:200, proteintech), CDK1 (1:200, abcam), CDK2 (1:200, proteintech), and Ki67 (1:1000, proteintech). Subsequently, the slides were incubated with secondary antibodies (Zsbio, Beijing, China) for 30 min. After staining with DAB, the results were visualized by Leica DM4B microscope.

### LUAD tumor cell derived xenograft model

All mice were treated humanely, and this study was approved by the Medical Experimental Animal Care Committee of Central South University. Lung adenocarcinoma A549 cells transfected with Vector/sh-CLSPN lentivirus and PC9 cells transfected with Vector/sh-CLSPN lentivirus were subcutaneously inoculated into the right flank of BALB/c mice (4 weeks, male, *n* = 5). Tumor size was detected every 3 days after injection and the tumor volume was calculated according to the formula: volume = 1/2 × (L × W^2^). All mice were sacrificed at day 21. Tumor tissues were separated and embedded with paraffin for IHC staining and analysis.

### Drug sensitivity analysis

The drug concentration and CLSPN expression profiles were downloaded from the CellMiner™ database (https:// discover.nci.nih.gov/ cellminer/) [25].

### Molecular docking

Two thousand one hundred fifteen FDA-approved drugs were obtained from ZINC database, which were divided into individual files and converted to PDBQT format using the Open Babel tool (http://openbabel.org/wiki/Main_Page) for virtual screening [26]. The 3D structure of CHK1 kinase domain in complex with a Claspin phosphopeptide (PDB ID: 7ako) downloaded from the PDB (https://www.rcsb.org/) was converted into PDBQT format using AutodockTools 1.5.7 [27]. Missing atoms were added by Swiss-PdbViewer (https://spdbv.unil.ch/) [28]. To finding receptor pockets in our complex, blind docking was conducted with the grid box 126 Å × 96 Å × 120 Å centered at 9.674, 14.053, and -1.22 to cover the whole structure. The semi-flexible molecular docking calculation function of AutoDock Vina 1.1.2 software was used to predict the binding affinities between the drugs and protein complex [29]. Fifty combined modes were computed for each drug. The lower the energy, the higher the affinity, and the stronger the binding force was between the drugs and the protein complex. The first five drugs with the lowest binding free energy were visualized by Pymol software 2.3, AutodockTools 1.5.7 and ProteinsPlus (https://proteins.plus).

#### Connectivity map analysis

We calculated connectivity map (CMap) score for the drugs predicted by molecular docking (darifenacin and eltrombopag were included in the small molecular library of CMap database). Briefly, we performed differential expression analysis between CSPLN high-expression samples and low-expression samples in TCGA-LUAD, SKCM, COAD, BRCA, CESC, PRAD and PAAD projects, whose corresponding cell lines were included in CMap database. The 300 differential expression genes (DEGs) with the most significant fold changes (top 150 up-regulated DEGs and top 150 down-regulated DEGs) were submitted to CMap website (https://clue.io) to conduct CMap analysis for each TCGA projects [30–32]. The CMap scores of the corresponding cell lines treated with darifenacin or eltrombopag for each TCGA projects were collected. Negative CMap score represented the gene expression pattern of the specific cell line treated with certain perturbation that was oppositional to the expression pattern of CSPLN high-expression group, which indicated this perturbation had the potential therapeutic efficacy for CSPLN high-expression group.

### Statistical analysis

The survival analyses in this study were determined by Kaplan–Meier curve and log-rank test. Spearman's or Pearson's test was used to conduct correlation analysis. Student's *t*-test was applied for comparison of two groups and one-way analysis of variance (ANOVA) was applied for comparison of multiple groups. *P*-value < 0.05 indicated statistical significance.

## Results

### Differential expression of CLSPN between tumor and normal tissue samples

**Fig. 1:**
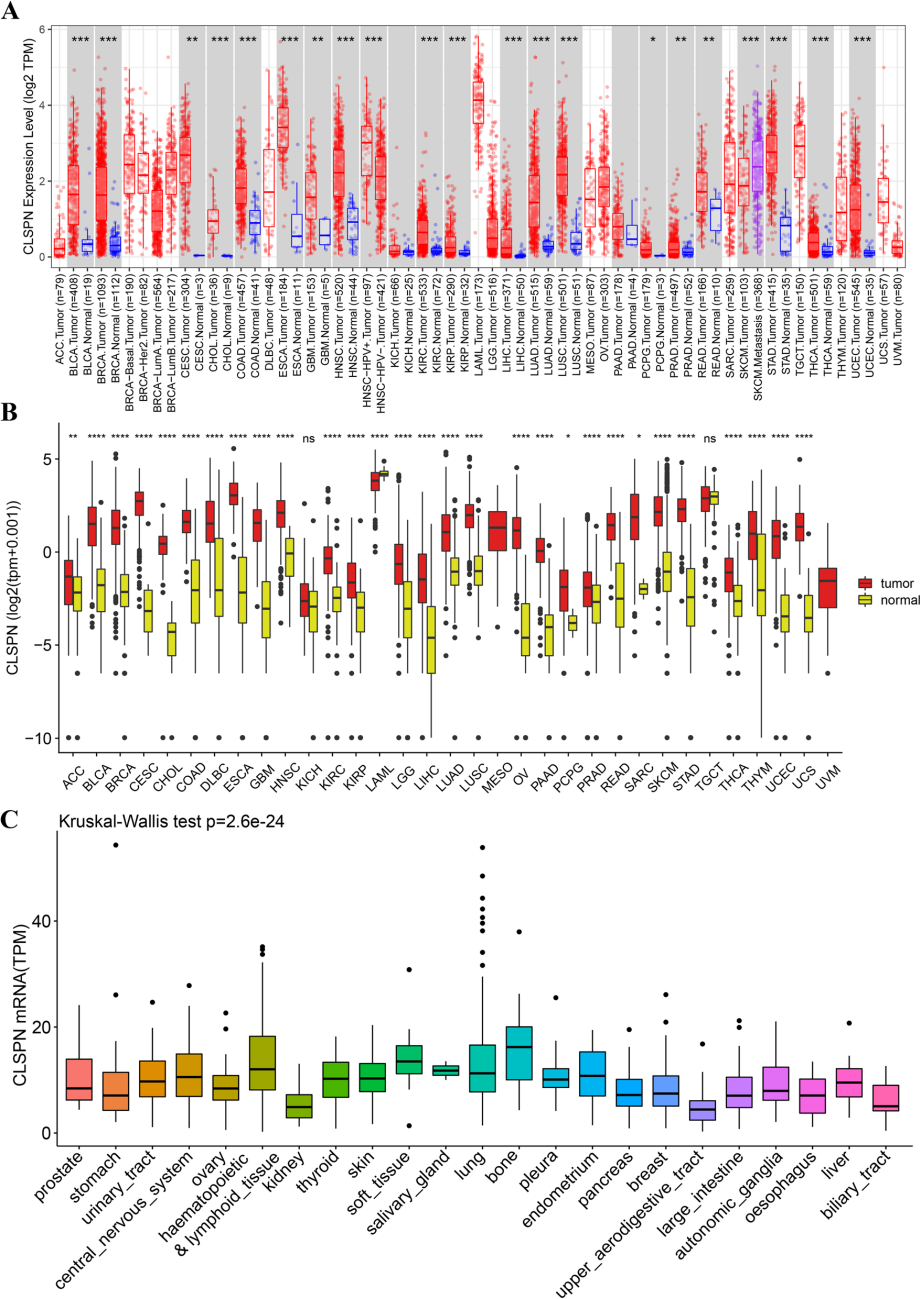
Differential expression of CLSPN in pan-cancer. **A** Expression levels of CLSPN in different TCGA tumors from TIMER database, *P < 0.05; **P < 0.01; ***P < 0.001. **B** Human CLSPN expression levels in different tumor types derived from the GTEx database (*P < 0.05, **P < 0.01, ***P < 0.001). **C** Expression levels of CLSPN in 23 tumor cell lines based on the CCLE datasets (Kruskal–Wallis test: *P* = 2.6e − 24)

Firstly, we compared CLSPN expression levels among tumors and matched normal tissues from 33 cancers using the TIMER database (Fig. 1A). Elevated CLSPN expression was observed in BLCA (bladder urothelial carcinoma), BRCA (breast invasive carcinoma), KIRC (kidney renal clear cell carcinoma), KIRP (kidney renal papillary cell carcinoma), LIHC (liver hepatocellular carcinoma), LUAD (lung adenocarcinoma), et al. The CLSPN expression abundances of various tissues in males and females were displayed in Supplementary Figure S1A and B. Overall, no gender difference was observed in the mRNA expression levels of CLSPN (Supplementary Figure S1C). The data from Human Protein Atlas (https://www.proteinatlas.org/) [32, 33] also suggested that CLSPN was highly expressed in multiple cancer samples (Supplementary Figure S1D).

Subsequently, we combined TCGA and GTEx datasets to further validate the CLSPN expression differences in multiple normal tissues and tumor tissues (Fig. 1B, *P* < 0.05). CLSPN was significantly upregulated in tumor tissues when compared with normal tissues. Furthermore, the CLSPN expression in diverse tumor cell lines was demonstrated with significant differences based on CCLE datasets (Fig. 1C, Kruskal–Wallis test: *P* = 2.6e − 24).

The results from the TCGA database were used to explore the correlation between CLSPN expression and clinicopathological stages in various cancers, which revealed the stage-specific expressional changes of CLSPN in some tumor types, such as BRCA, KICH (Kidney Chromophobe), KIRC, LIHC, LUAD, et al. (Supplementary Figure S2A-T).

### Prognostic value of CLSPN across cancers

**Fig. 2:**
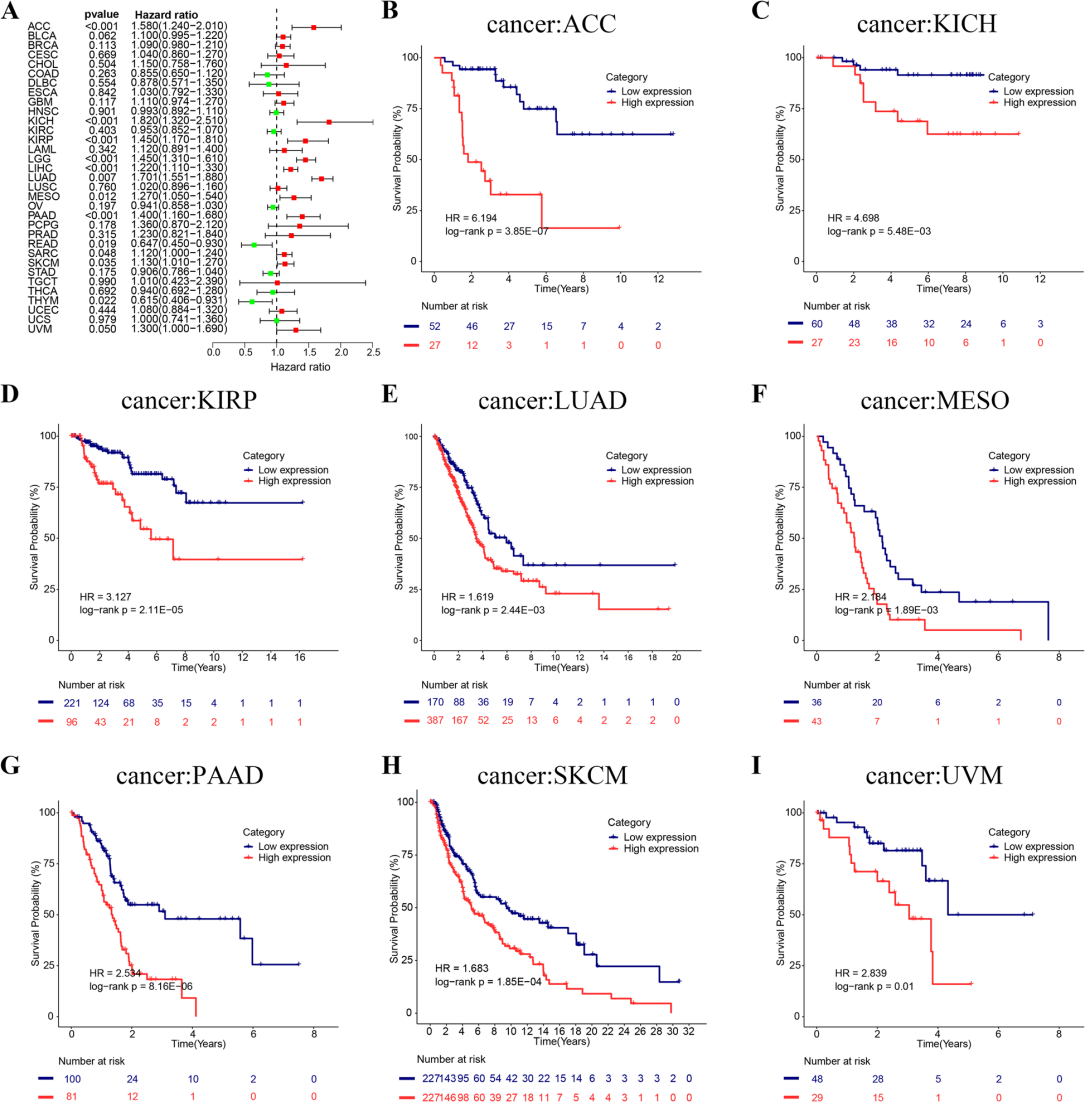
Association between the CLSPN expression and the OS of cancer patients. **A** Forest plot displaying the effect of CLSPN expression on OS across 33 types of cancer using Cox regression model. **B**-**I** Kaplan–Meier survival curves of the correlations between the CLSPN expression and OS. A red line represents high CLSPN expression, and the blue lines represent low CLSPN expression (*P* < 0.05 indicated statistical significance)

We further conducted survival analysis in different cancer types to investigate the prognostic value of CLSPN. The results of Cox proportional hazards model demonstrated that CLSPN was significantly connected with OS in most cancers (Fig. 2A, *P* < 0.05). Kaplan–Meier survival curves revealed that high CLSPN expression was obviously related to poor OS in ACC (Adrenocortical carcinoma), KICH, KIRP, LUAD, MESO (mesothelioma), PAAD (pancreatic adenocarcinoma), SKCM (skin cutaneous melanoma), UVM (uveal melanoma) (Fig. 2B-I). The GEO (Gene Expression Omnibus) dataset further validated the influence of CLSPN on the prognosis of tumor patients in clinical cohort (Supplementary Figure S3A-I).

Furthermore, the result of DSS analysis indicated CLSPN expression was correlated with patients prognosis (Supplementary Figure S4A). Kaplan–Meier survival analysis showed an association between CLSPN and poor prognosis in ACC, KICH, KIRP, LIHC, LUAD and PAAD patients (Supplementary Figure S4B-G, *P* < 0.05). Cox regression analysis of the DFI revealed that the increased CLSPN expression was a risk factor in KIRP, LIHC, LUAD, and PAAD (Supplementary Figure S4H, *P* < 0.05). A significant association were presented by KM survival analysis (Supplementary Figure S4I-N, *P* < 0.05). With regard to PFI, CLSPN expression level was related to the ACC, BLCA, KICH, KIRP, LIHC and LUAD patients' prognosis (Supplementary Figure S5A, *P* < 0.05). The KM survival analysis results were presented in Supplementary Figure S5B-I.

### Genetic alteration analysis

**Fig. 3:**
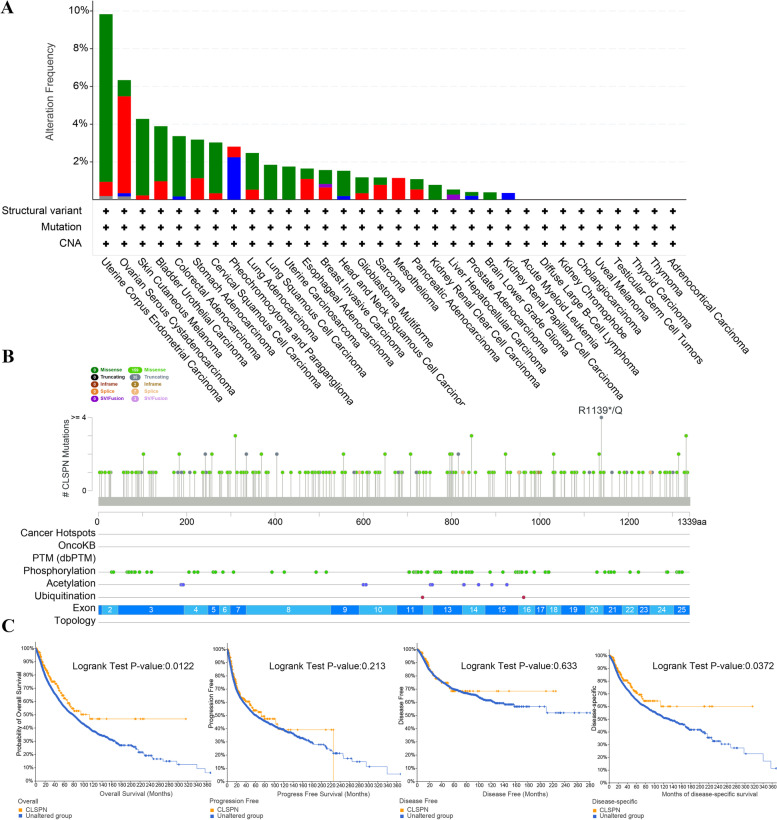
Mutation feature of CLSPN in TCGA tumors obtained from the cBioPortal tool. **A** Alteration frequency with the mutation type of CLSPN in human pan-cancer. **B** Mutation sites of CLSPN are displayed. **C** The correlation between CLSPN mutation status and OS, PFS, DFS and DSS in pan-cancer (*P* < 0.05 indicated statistical significance)

We analyzed various tumor samples to explore the genetic alteration status of CLSPN. As shown in Fig. 3A, the highest alteration frequency of CLSPN (> 8%) was observed in patients with uterine endometrial tumors with “mutation” as the primary type. The types, sites and case numbers of the CLSPN genetic alteration were further presented in Fig. 3B. Moreover, we analyzed the potential correlation between genetic alteration of CLSPN and prognosis of cases with distinct cancer types. The result revealed that altered CLSPN had better prognosis in OS (*P* = 0.0122) and DSS (*P* = 0.0372) but not in PFS (*P* = 0.213) and DFS (*P* = 0.613), in comparison with patients without CLSPN alterations (Fig. 3C).

### Relationship between CLSPN expression and the tumor microenvironment

**Fig. 4:**
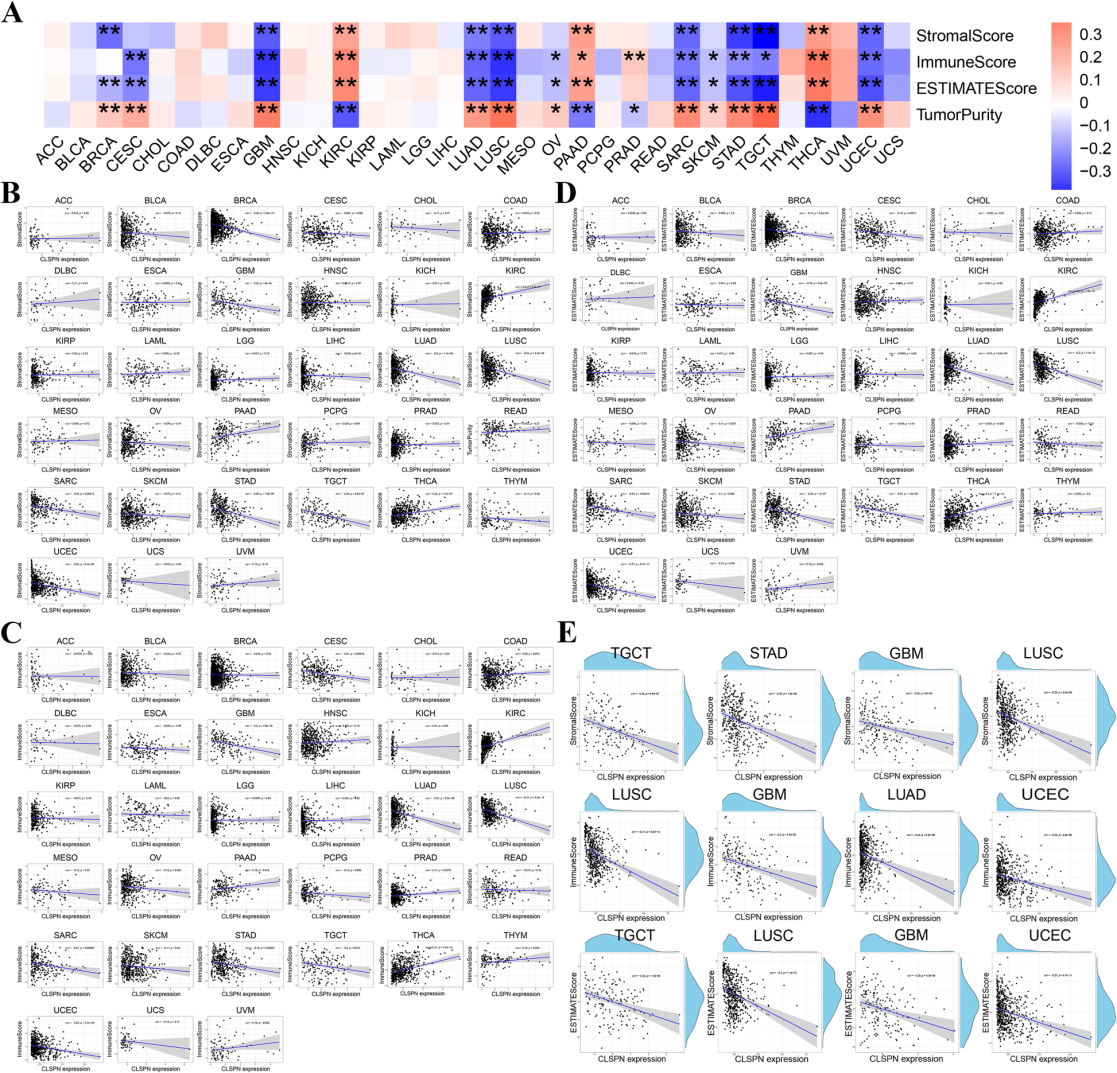
Association of CLSPN expression with StromalScore, ImmuneScore and ESTIMATEScore in pan-cancer. **A** The heatmap of the relationship between CLSPN expression and StromalScore, ImmuneScore, ESTIMATEScore and TumorPurity. **B** Correlation of CLSPN expression with StromalScore. **C** Correlation of CLSPN expression with ImmuneScore. **D** Correlation of CLSPN expression with ESTIMATEScore. **E** Top 4 cancers significantly related to CLSPN expression by ImmuneScore, StromalScore, and ESTIMATEScore, respectively (*P* < 0.05 indicated statistical significance)

Numerous studies demonstrated that tumor immune microenvironment had an impact on the cancer therapeutic effectiveness. Accordingly, we further investigated the correlation between TME and CLSPN expressions using the ESTIMATE algorithm across 33 cancer types (Fig. 4A). CLSPN was significantly negatively associated with StromalScore, ImmuneScore and ESTIMATEScore in LUAD, LUSC, etc. (Fig. 4B - D). The top 4 tumors were most significantly related to CLSPN expression in StromalScore, ImmuneScore, and ESTIMATEScore were presented in Fig. 4E.

**Fig. 5:**
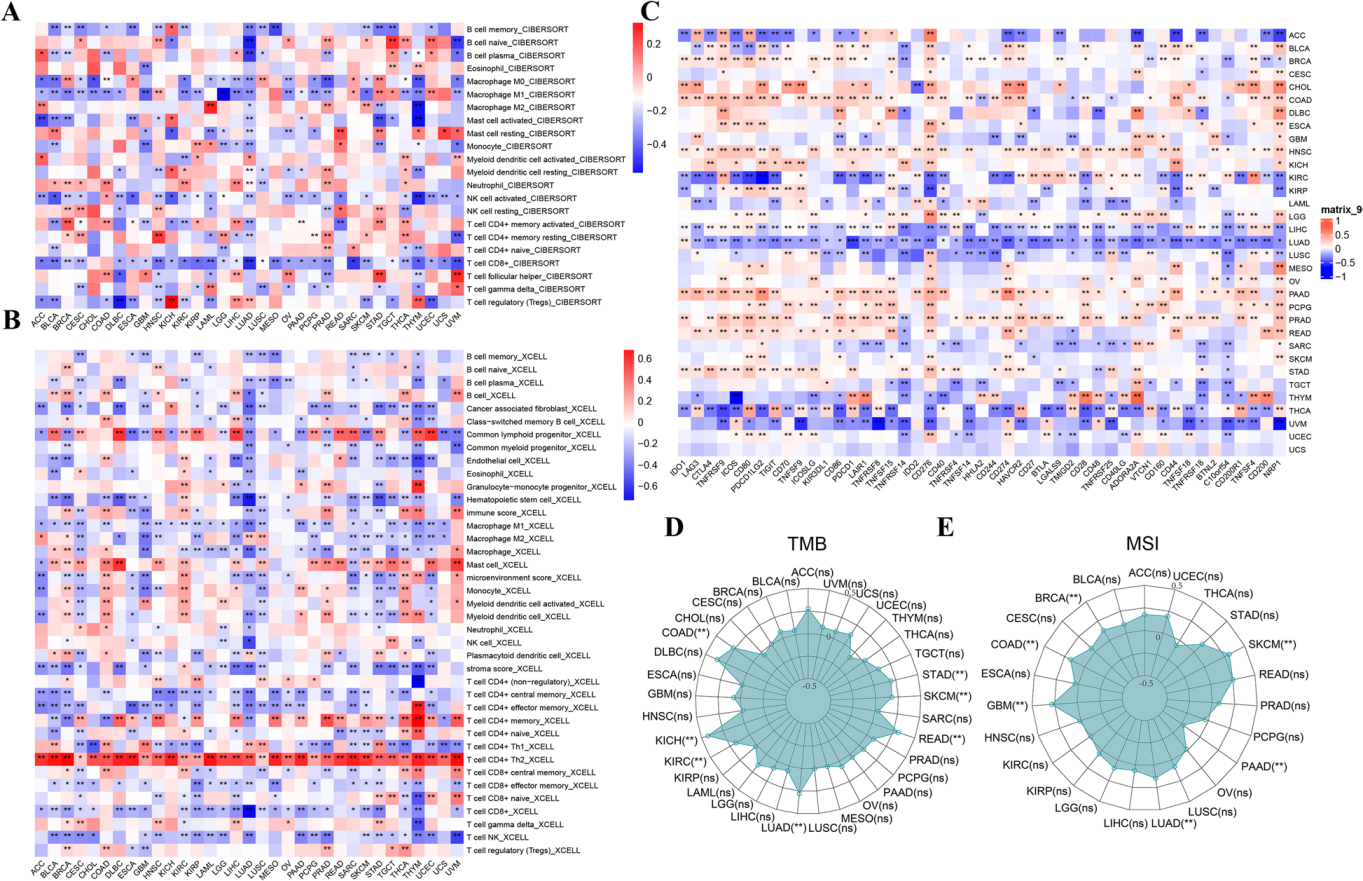
The correlation between CLSPN expression and immunity, TMB, MSI in different cancer types. **A** The correlation between CLSPN expression and immune cell infiltration across all tumors in TCGA examined by the CIBERSORT database. **B** The correlation between CLSPN expression and diverse immune cells infiltration in pan-cancer based on X-Cell database. **C** The heatmap of the correlation between 47 immune checkpoint genes and CLSPN expression. **D** Radar map of the correlation between TMB and CLSPN expression. **E** Radar map of the correlation between MSI and CLSPN expression. *P < 0.05, **P < 0.01, ***P < 0.001

Algorithms of CIBERSORT and XCELL were applied to analyze the correlation of infiltrating immune cells and CLSPN expression in various cancers. In most cancer types, CLSPN expression and number of infiltrating CD8 + T cells showed a negative correlation, as depicted in Fig. 5A and B. Furthermore, CLSPN expression was associated with 47 immune checkpoint genes in LUAD, 37 in PRAD, and 42 in LIHC (Fig. 5C). These results suggested that CLSPN expression alteration may reflect tumor immunity level.

It has been reported that TMB and MSI are biomarkers of immune response of tumors. As shown in Fig. 5D, CLSPN notably correlated with TMB in several tumors, such as KICH, LUAD and READ. CLSPN was positively associated with the MSI in GBM, COAD, BRCA, SKCM and LUAD tissues (Fig. 5E).

### Correlation of CLSPN expression with MMR gene and DNA methylation

**Fig. 6:**
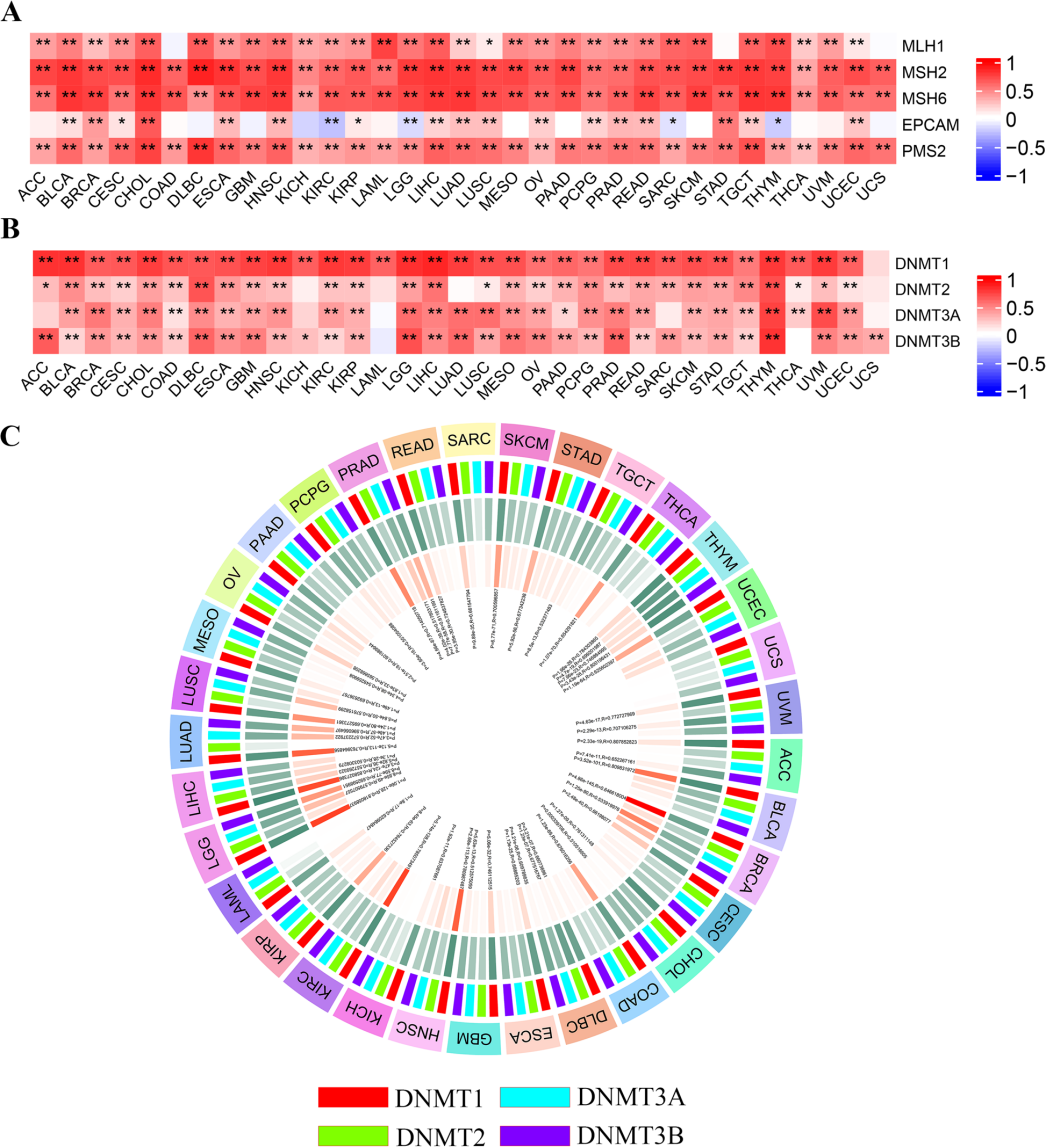
Correlation analysis between CLSPN expression and five MMR genes and four DNA methyltransferases in pan-cancer. **A** The heatmap of association between CLSPN expression and five MMR genes (MLH1, MSH2, MSH6, EPCAM, PMS2). **B** The heatmap of correlation between CLSPN expression and the expression of four methyltransferases (DNMT1, DNMT2, DNMT3A, DNMT3B). **C** Spearman’s correlation analysis of CLSPN expression with four DNA methyltransferases across 33 cancers. *P < 0.05, **P < 0.01, ***P < 0.001

To investigate whether CLSPN expression could predict tumor progression, we selected five typical MMR genes, and evaluated their association with CLSPN*.* DNA mismatch repair genes were highly associated with the CLSPN expression in almost all cancer types (Fig. 6A).

In addition, the relationships between CLSPN and four methyltransferases were also observed in the majority of cancer types (Fig. 6B and C). The correlation between CLSPN expression and CLSPN methylation was presented in Supplementary Figure S6A. We further evaluated the impact of single CpG on LUAD prognosis in MethSurv using TCGA data. As shown in Supplementary Figure S6B, the hyper methylation of CLSPN-body-Island-cg00463507 (HR = 1.433, *P* = 0.046), CLSPN − TSS200 − Island − cg04263115 (HR = 1.502, *P* = 0.02), CLSPN − TSS1500 − Island − cg10246273 (HR = 1.435, *P* = 0.025) indicated poorer OS in TCGA LUAD patients. However, the hyper methylation of CLSPN − 5'UTR;1stExon − Island − cg25109252 (HR = 0.662, *P* = 0.013) suggested a good OS. The heatmap suggested that cg02106385 of CLSPN displayed the highest level of DNA methylation in LUAD (Supplementary Figure S6C). Kaplan–Meier survival analysis was used to assess the relationship between promoter methylation of CLSPN and prognosis of patient (Supplementary Figure S7A—B)*.*

### Correlation between CLSPN expression and stemness score in pan-cancer

The stemness index correlated with tumor pathology and could be used to predict clinical prognosis. We explored whether CLSPN expression was related with stemness score in a variety of cancers by conducting a correlation analysis (Supplementary Figure S8A). The result indicated that CLSPN was positively associated with mRNAsi in ACC, BLCA, LUAD, etc. and mDNAsi in BRCA, CESC, LUAD, etc. (Supplementary Figure S8B). The top 6 tumors most positively correlated with mRNAsi and mDNAsi were presented in Supplementary Figure S8C.

### The distribution of CLSPN in LUAD at single-cell level

We evaluated the expression of CLSPN in LUAD patients at the single-cell level using three datasets (NSCLC_EMTAB6149, NSCLC_GSE127465 and NSCLC_GSE143423) from TISCH database. The distribution of CLSPN expression in databases was presented in Supplementary Figure S9A. In NSCLC_EMTAB6149, 12 cell types were found. The result suggested that CLSPN was mainly expressed at the CD8Tex and malignant cells (Supplementary Figure S9B). In NSCLC_GSE127465, CLSPN was mainly distributed in CD8Tex, NK, DC, Mona/Macro, Mast, Neutrophils and malignant cells (Supplementary Figure S9C). In NSCLC_GSE143423, CLSPN was mainly concentrated in CD8T, Mona/Macro and malignant cells (Supplementary Figure S9D). These results indicated that CLSPN may function in tumor immune microenvironment.

### CLSPN was upregulated in LUAD tissues and cell lines

**Fig. 7:**
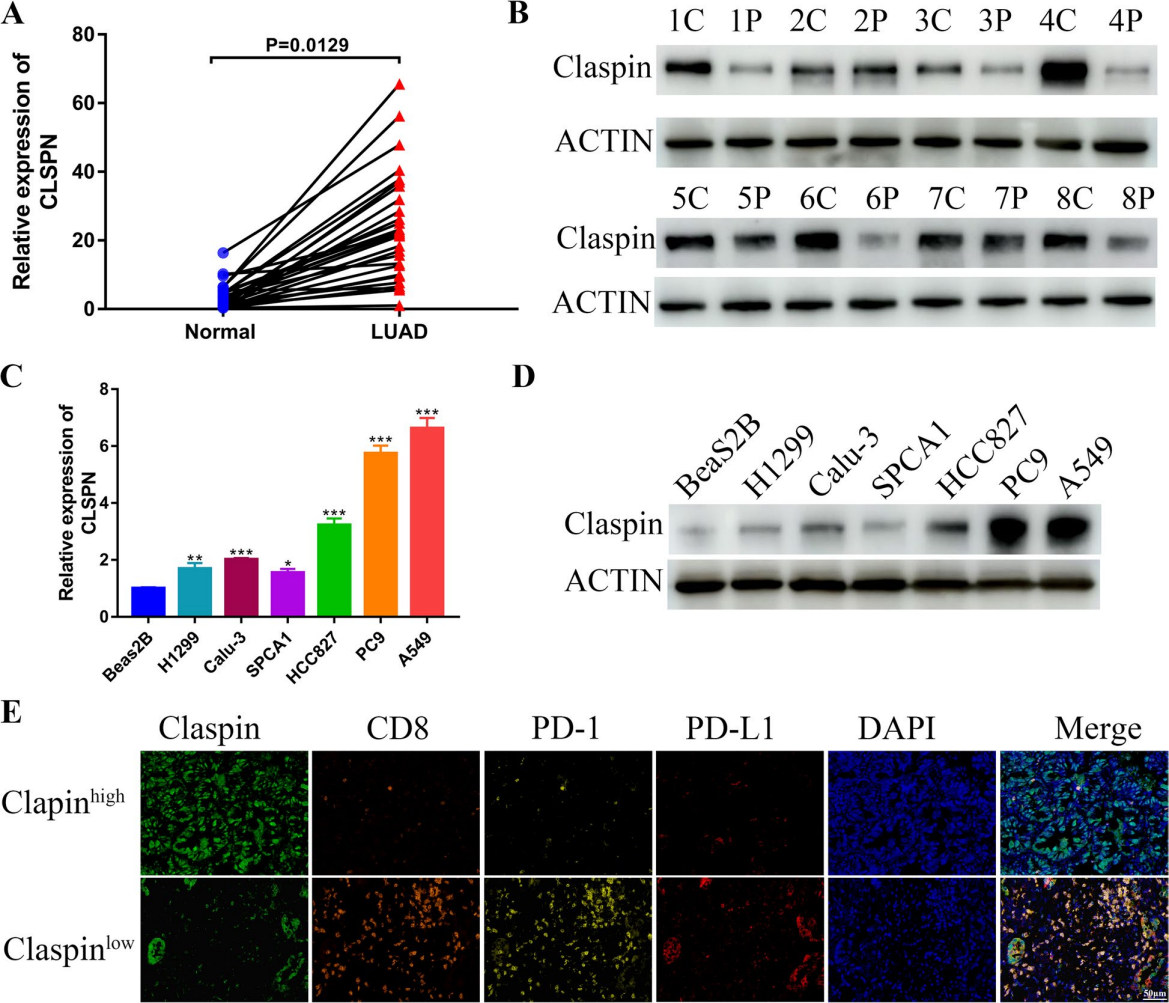
Identification of CLSPN expression in LUAD tissues and cell lines. **A** CLSPN mRNA expression in LUAD tissues and adjacent normal lung tissues (*n* = 28). **B** Claspin protein levels in paired tissues (*n* = 8). **C** CLSPN mRNA levels in 6 LUAD cell lines (H1299, Calu-3, SPCA1, HCC827, PC9 and A549) and human normal pulmonary epithelial cell (Beas2B). **D** Claspin protein levels in 6 LUAD cell lines and human normal pulmonary epithelial cell. **E** Representative immunofluorescence staining of Claspin, CD8, PD-1, and PD-L1 in high- and low-Claspin group (Scare bar = 50 μm). *P < 0.05, **P < 0.01, ***P < 0.001

Based on the bioinformatics analysis, we further evaluated the role of CLSPN *in* LUAD. The expression level of CLSPN mRNA and Claspin protein in LUAD tissues was significantly higher than that in adjacent normal tissues (Fig. 7A and B). The expression of CLSPN in Beas2B cell line (Normal pulmonary epithelial cell) and 6 human lung cancer cell lines was detected by RT-qPCR. CLSPN expression was significantly increased in lung cancer cell lines, especially in PC9 and A549 cell lines (Fig. 7C and D). We further explored the correlation between Claspin and immune infiltration. The immunofluorescence staining result suggested that Claspin was remarkably negatively associated with CD8 + T cell infiltration and immune checkpoints including PD-1 and PD-L1 (Fig. 7E), which confirmed the result of bioinformatics analysis above.

### Knockdown CLSPN suppressed the LUAD cells proliferation

**Fig. 8:**
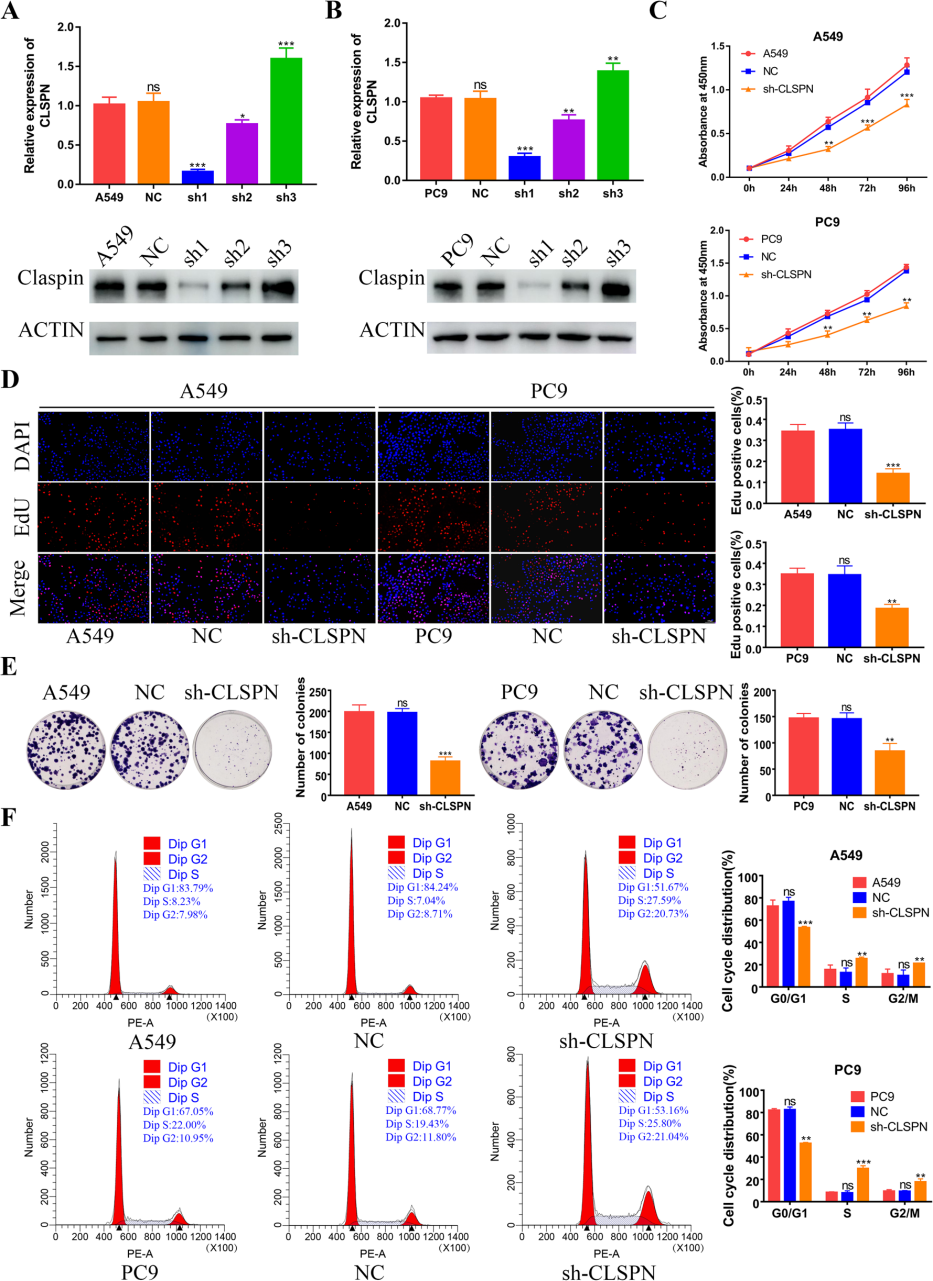
Knockdown CLSPN significantly inhibited lung cancer cell proliferation and induced S and G2/M arrest in vitro. **A** RT-qPCR and western blot confirmed the knockdown efficiency of CLSPN in A549 cells. **B** The knockdown efficiency of CLSPN in PC9 cells. **C** The effect of CLSPN on cell viability was confirmed by CCK-8 assays. **D** EdU assays were used to evaluate cell proliferative ability after knockdown CLSPN (Scare bar = 100 μm). **E** Colony formation in untreated, NC and sh-CLSPN groups. **F** Cell cycle analyses were performed by flow cytometry. *P < 0.05, **P < 0.01, ***P < 0.001, *ns*: no significance

To further investigate the function of CLSPN in LUAD, we selected A549 cells and PC9 cells which was with highest CLSPN expression for further functional study. Then, we constructed 3 shRNA to knockdown CLSPN in A549 and PC9 cells. RT-qPCR and Western blot analysis was performed to evaluate the knockdown efficiency of CLSPN (Fig. 8A and B). The results of CCK8 assays (Fig. 8C), EDU (Fig. 8D) and colony formation assays (Fig. 8E) indicated that the knockdown of CLSPN inhibited the proliferative activity of LUAD cells. Compared with negative control, knockdown CLSPN resulted in S and G2/M arrest in A549 and PC9 cells (Fig. 8F).

### Knockdown CLSPN suppressed cell cycle signal both in vitro and in vivo

**Fig. 9:**
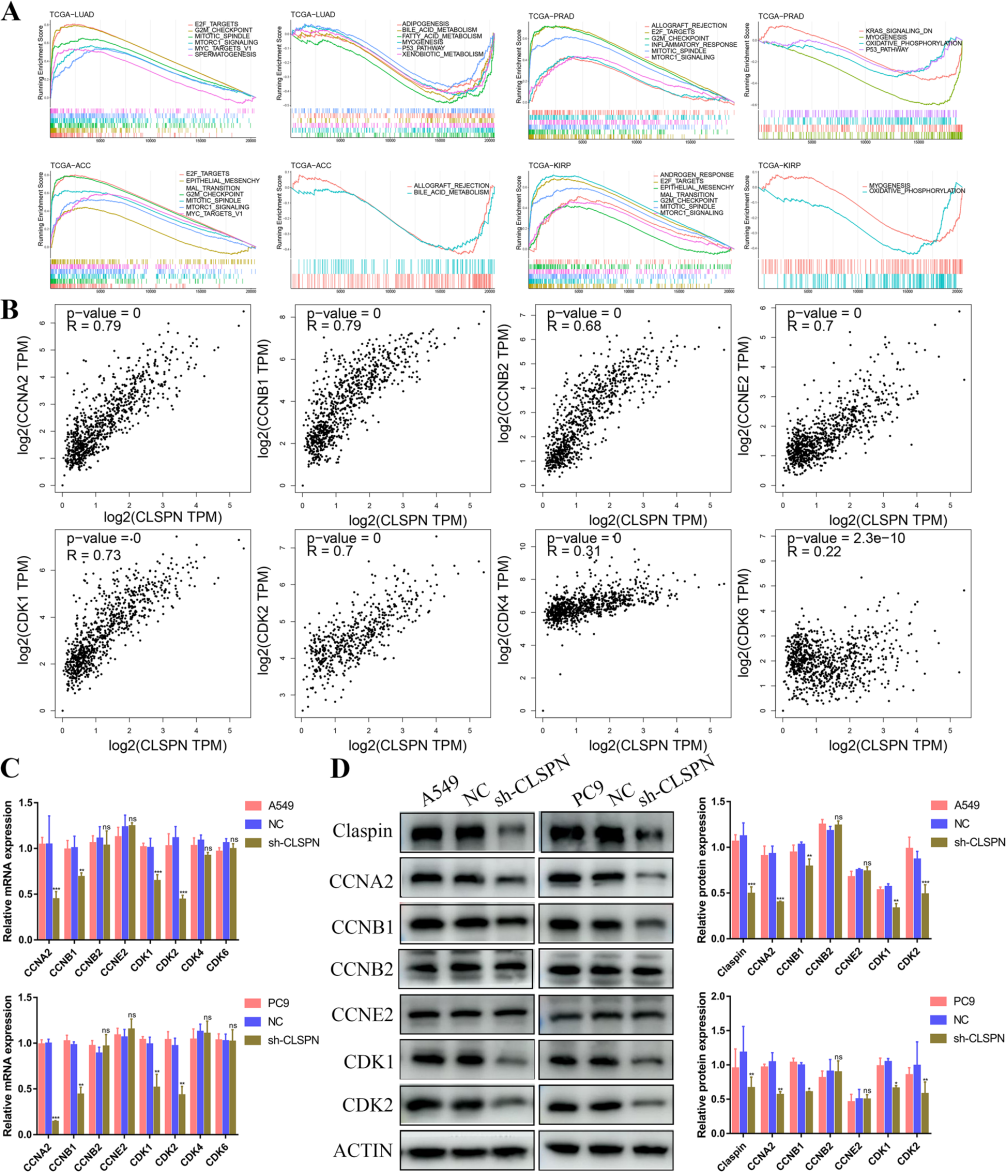
CLSPN associated with cell cycle signal. **A** Functional enrichment analysis of CLSPN through GSEA. **B** The correlation between CLSPN expression and CCNA2, CCNB1, CCNB2, CCNE2, CDK1, CDK2, CDK4 and CDK6 in the GEPIA2.0 database. **C** RT-qPCR validated the mRNA expression of CCNA2, CCNB1, CCNB2, CCNE2, CDK1, CDK2, CDK4 and CDK6 after knockdown CLSPN. **D** Knockdown CLSPN significantly inhibited the protein expression of CCNA2, CCNB1, CDK1 and CDK2 in A549 and PC9 cells. *P < 0.05, **P < 0.01, ***P < 0.001, *ns*: no significance

The functional enrichment analysis in Fig. 9A suggested that CLSPN was remarkably associated with cell cycle in LUAD. We further explored the correlation between CLSPN and Cyclin-dependent kinase (CDK) family and Cyclin family expression. The results demonstrated that CLSPN was positively correlated with the expression of CCNA2, CCNB1, CCNB2, CCNE2, CDK1, CDK2 (Fig. 9B). The expression of CCNA2, CCNB1, CDK1 and CDK2 were significantly decreased at the mRNA and protein level after knockdown CLSPN in A549 and PC9 cells (Fig. 9C and D).

**Fig. 10:**
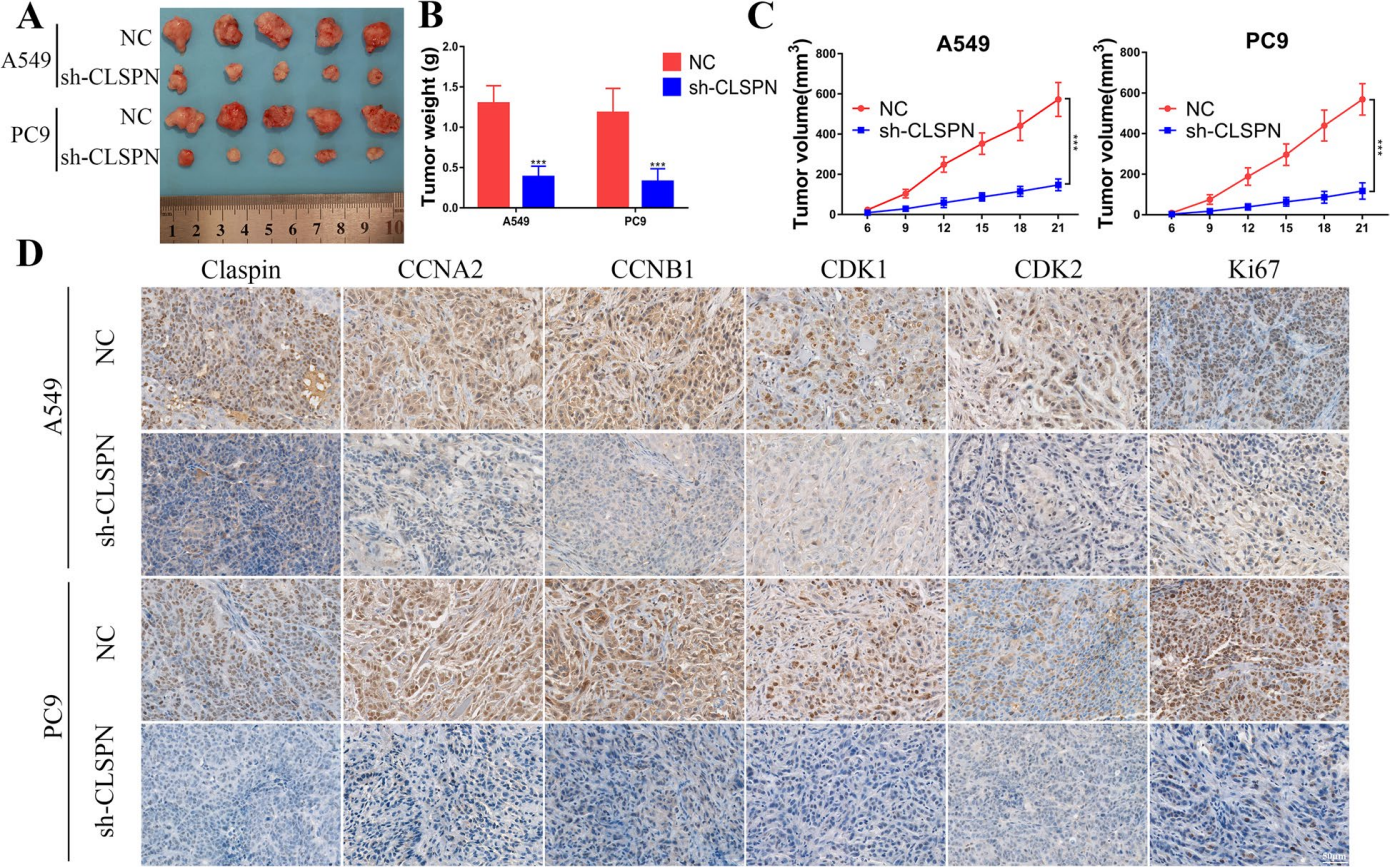
Knockdown CLSPN suppressed LUAD cells growth in vivo. **A** A549 cells or PC9 cells with different CLSPN expression levels were subcutaneously inoculated in the right flank of BALB/c mice (5 × 10^6^ cells/mouse; *n* = 5 in each group), and then the tumor volume was calculated using the following formula: V (mm^3^) = (L × W.^2^) × 0.5 (L: tumor length, W: width). The mice were sacrificed at day 21 after subcutaneous implantation. The formation of tumor masses was presented. **B** The histogram displayed the tumor weight in different groups. **C** Tumor size was measured every 3 days until day 21 (Data were represented as the mean ± SD). **D** Immunohistochemistry staining for Claspin, CCNA2, CCNB1, CDK1, CDK2 and Ki67 in subcutaneous tumors (Scare bar = 50 μm). ***:* P* < 0.001

To explore the function of CLSPN on LUAD growth in vivo, we constructed LUAD xenograft mouse models by subcutaneously injecting A549 and PC9 cells with CLSPN—RNAi or vector lentivirus stably transduction in the right flank of BALB/c mice. The volume and weight of xenografts in sh-CLSPN group was lower than that in NC group (Fig. 10A and B). Knockdown CLSPN significantly reduced the tumor growth rate in A549 and PC9 cells (Fig. 10C). The immunohistochemistry result of subcutaneous tumor indicated that knockdown CLSPN significantly down regulated CCNA2, CCNB1, CDK1, CDK2 and Ki67 expression (Fig. 10D).

### Drug sensitivity analysis of CLSPN

Next, we analyzed the data from CellMiner™ to investigate the IC50 values of anti-cancer drugs and CLSPN expression. We discovered that CLSPN expression was positively associated with the drug sensitivity of PF − 06463922, salinomycin, KU − 55933, Olaparib, AZD − 3463, etc. and negatively associated with sensitivity of Birinapant, 6 − Thioguanine, 6 − THIOGUANIN and Nelarabine (Supplementary Figure S10).

### Validation of the affinity of the candidate drugs by molecular docking analysis and CMap analysis

**Fig. 11:**
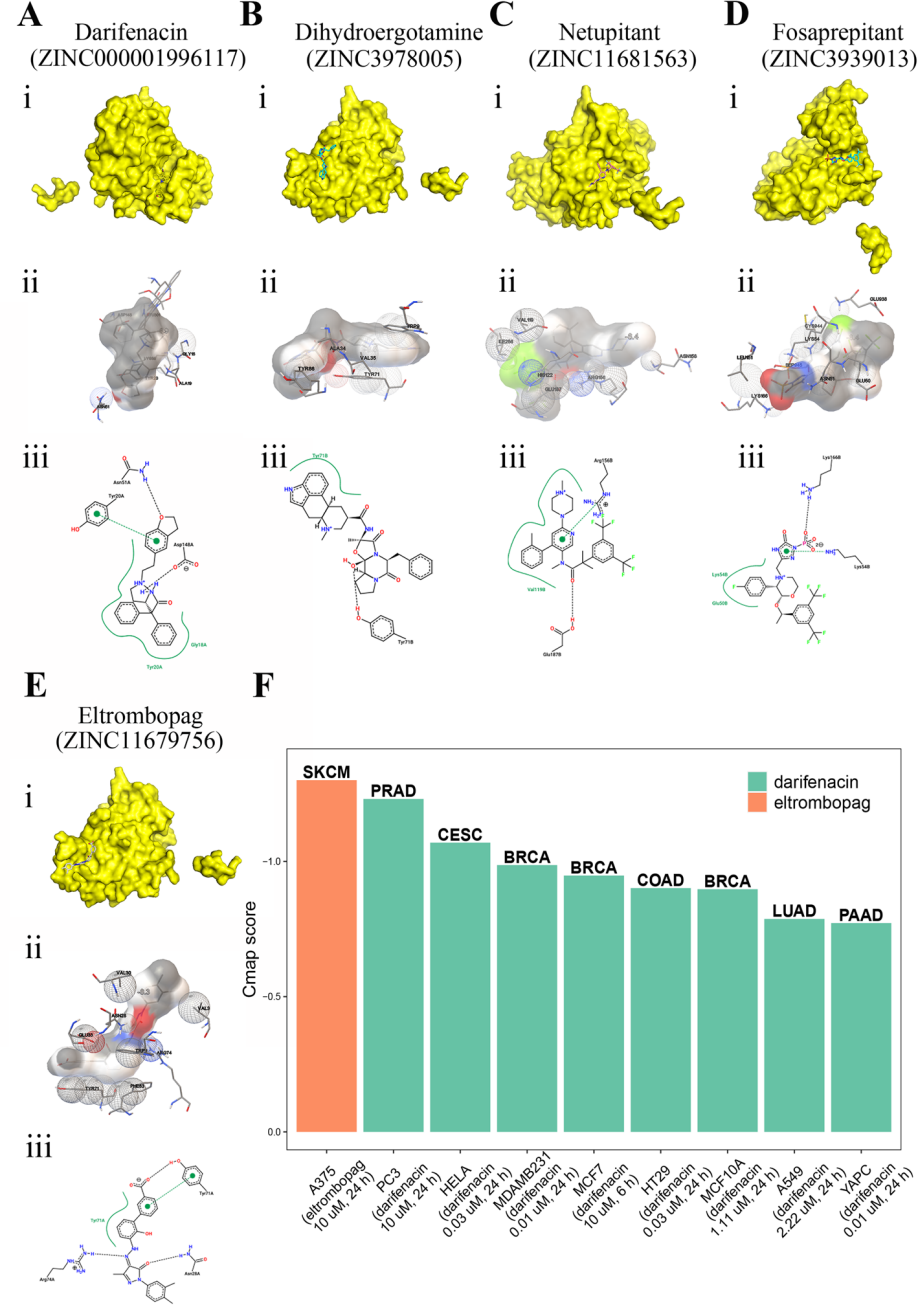
Validation of the affinity of the candidate drugs by molecular docking analysis and CMap analysis. **A** Binding mode of the protein complex and Darifenacin. **B** Binding mode of the protein complex and Dihydroergotamine. **C** Binding mode of the protein complex and Netupitant. **D** Binding mode of the protein complex and Fosaprepitant. **E** Binding mode of the protein complex and Eltrombopag. (i) The binding sites of drugs in the 3D structure of the protein complex were displayed by PyMOL software. (ii) AutoDockTools showed the interaction between the protein complex and drugs. (iii) 2D interactions of compounds and their targets. The directional bonds between the protein complex and ligands were drawn as dashed lines, and the interacting protein complex residues and ligands were visualized as structural diagrams. Hydrophobic contact was represented by the spline part, highlighting the interaction between the hydrophobic part of the ligand and the label of contacting amino acid. **F** CMap analysis to validate the drugs predicted by molecular docking in diverse cancers

Claspin was discovered as an adaptor or scaffold protein necessary for Chk1 activation in response to DNA replication blocks and stalled replication forks [2]. Previous researches reported that the repetitive phosphopeptide motif in human Claspin was important for Claspin-Chk1 interaction to mediate Claspin function [34, 35]. Considering that Claspin acted as a scaffold protein facilitating the recruitment of CHK1 into larger complexes, we used the structure of CHK1 kinase domain and Claspin phosphopeptide complex (PDB ID:7ako) for molecular docking to screen related FDA-approved drugs. The results indicated that the drugs bound to the complex of Claspin phosphopeptide and CHK1 mainly through strong electrostatic and hydrogen-bonding interactions (Fig. 11A-E). The first five drugs with the lowest binding energy were Darifenacin, Dihydroergotamine, Netupitan, Fosaprepitant and Eltrombopag. The binding energy were − 8.9 kcal/mol, − 8.8 kcal/mol, − 8.8 kcal/mol, − 8.4 kcal/mol, − 8.4 kcal/mol indicating a highly stable binding. We performed CMap analysis to validate the drugs predicted by molecular docking, which indicated that eltrombopag might serve as a potential therapeutic drug in SKCM patients with highly expressed CLSPN, and darifenacin might act as potential therapeutic drug in PRAD, CESC, BRCA, COAD, LUAD, PAAD patients with CLSPN high expression (Fig. 11F).

## Discussion

Recently, CLSPN has been reported to associate with tumorigenesis, tumor metastasis and therapeutic resistance. Upregulated CLSPN was observed in patients with prostate cancer [8], renal cell carcinoma [10], gastric cancer [36], melanoma [37], anaplastic thyroid cancer [38] and other malignant diseases or animal models [39–41]. However, a multi-omics pan-cancer analysis on the function of CLSPN from the comprehensive angle has not been reported yet. Our study found out that CLSPN was significantly overexpressed in different tumor tissues, and the upregulated expression of CLSPN was closely associated with stage and clinical outcomes of human cancers. In our research, we found by bioinformatics that CLSPN was significantly upregulated and has a close relationship with the prognosis of LUAD, which was further verified by in vivo and in vitro experiments. Knockdown CLSPN significantly inhibited cancer cell growth both in vitro and *vivo* experiments. GSEA and correlation analysis suggested that CLSPN was associated with cell cycle signal. Knockdown of CLSPN downregulated expression of CCNA2, CCNB1, CDK1 and CDK2 at both mRNA and protein level.

Accumulated evidence testified that CLSPN variants were correlated with susceptibility to cancers as well as sporadic tumorigenesis [42–44]. To thoroughly understand the features of CLSPN, we further explored its alterations utilizing cBioPortal database. Among these alterations, we observed missense mutation as the commonest type, while inframe mutation was the rarest, which was consistent with the research of other scholars [44, 45]. Besides, our results revealed that the truncate mutation of R1139*/Q site in CLSPN had the highest frequency, which had never been proved by basic experiments before. These findings may offer new insight for genetic alterations analysis of CLSPN.

Immunotherapy represented by immune checkpoint blockade (ICB) and adoptive cell therapy (ACT) had been rapidly developed in the past decades, part of which had been applied to clinical practice and achieved remarkable effects [46]. Although immunotherapy brought about the gospel for advanced malignant tumor patients with multidrug resistance, increasing evidence indicated that there were still a variety of challenges in its successful application [47, 48]. TME (Tumor Microenvironment) was an extremely complex structure mainly composed of tumor cells, stromal cells, immune cells, cancer-associated fibroblasts (CAFs), blood vessels, extracellular matrix and various signal molecules [49]. After persistent tumor antigens stimulation, the exhausting and remodeling of relevant effector cells in the TME led to immune escape of tumors and eventually facilitated tumor progression [50, 51]. Therefore, the identification of new predictive biomarkers and the complete understanding of immune infiltration status in cancer patients were especially significant for the selection of accurate individualized immunity treatment. Currently, whether CLSPN could influence tumor immune microenvironment remained unknown. In our study, we found for the first time that CLSPN expression had a close relationship with immune cell infiltration among multiple tumor types. As for LUAD, CLSPN level was negative associated with CD8 + T cells, CD4 + T cells, NK cells, and macrophage cell infiltration. Our experiments also demonstrated a negative correlation between CLSPN and CD8 + T-cell infiltration, PD-1 and PD-L1 expression in LUAD. Furthermore, our enrichment analyses indicated that CLSPN was strongly associated with inflammatory response and interferon gamma (IFN-γ) response. IFN-γ was a double-edged sword for tumor immunotherapy [52]. On the one hand IFN-γ could recruit T cells, NK cells, and NKT cells to tumors through releasing CXCL9, CXCL10, and CXCL11 chemokines; on the other hand, IFN-γ had been proved to recruit Treg cells to avoid immune recognition and upregulate immunosuppression factor (PDL1, IDO1, FAS and FASL) to promote carcinogenesis [53, 54]. These results suggested that CLSPN affected the infiltration of immune cells in tumor tissue through immune—regulatory cytokines. The clarification of the interaction mechanism between CLSPN and TME may propose a new target for immunotherapy.

At present, the role of CLSPN in therapeutic resistance has gradually aroused attention. Previous studies had reported that CLSPN overexpression promoted docetaxel and radiation resistance in cancer patients [8, 55]. Our study probed the potential correlation between CLSPN expression and IC50 values of anti-cancer compounds in different human cancer cell lines, and found out that CLSPN expression was positively associated with drug sensitivity of PF − 06463922, salinomycin, KU − 55933, Olaparib, AZD − 3463, etc. and negatively correlated with drug sensitivity of Birinapant, 6 − Thioguanine, 6 − THIOGUANIN, Nelarabine. Notwithstanding, more clinically evidence needs to be further provided to assess the influence of these drugs on tumor therapy.

Although our study is the first to comprehensively unveil the polytrophic functions of CLSPN in cancer, there is not without limitations. Because this study mainly focuses on the correlation analysis between CLSPN and prognostic value, mutation status, immune cell infiltration, and drug sensitivity in diverse human cancer types by bioinformatics database, the molecular mechanisms of CLSPN in tumor immunity require further verifications in the future. As the sequencing data about CLSPN are collected from diverse databases, our analysis may involve systematic bias. Therefore, we should spare more efforts to investigate the specific role of CLSPN in different cancers.

## Conclusion

Overall, our study revealed that CLSPN might function as a potential tumor biomarker in most cancers, especially in LUAD. Moreover, CLSPN was associated with immune cell infiltration in cancers, providing a potential therapy target for future cancer treatment.

## Supplementary Information

**Additional file 1: Table S1.** Clinicopathological characteristics of LUAD cohort in Xiangya Hospital.

**Additional file 2: Figure S1.** CLSPN expression in different tissues. (A & B) The CLSPN enrichments of different tissues in males and females were displayed by TCGA and GTEx database. (C) The mRNA expression levels of CLSPN in different genders. (D) The mRNA and protein expression of CLSPN in cancers downloaded from Human Protein Atlas.

**Additional file 3: Figure S2**. Association between CLSPN expression and tumor stage in (A) BRCA; (B) COAD; (C) KICH; (D) KIRC; (E) KIRP; (F) LIHC; (G) LUAD; (H) LUSC; (I) MESO;(J) BLCA; (K) CHOL; (L) ESCA; (M) HNSC; (N) PAAD; (O) READ; (P) SKCM; (Q) STAD; (R) TGCT; (S) THCA; (T) UVM. *P < 0.05, **P < 0.01, and ***P < 0.001.

**Additional file 4: Figure S3.** Kaplan–Meier analysis of the correlation between CLSPN expression and the OS of cancer patients with (A) ACC, (B) KICH, (C) PRAD, (D) PAAD, (E) LUAD, (F) BLCA, (G) SARC, (H) READ and (I) COAD in GEO clinical cohort. A red line represents high CLSPN expression, and the blue lines represent the low CLSPN expression.

**Additional file 5: Figure S4.** Association between CLSPN expression levels and disease-specific survival (DSS) and disease-free interval (DFI) of cancer patients. (A) Forest plot of the association of CLSPN expression and DSS in 33 types of tumor. (B–G) Kaplan–Meier analysis of the association between CLSPN expression and DSS. (H) Forest plot of the association of CLSPN expression and DFI in 33 types of tumor. (I–N) Kaplan–Meier analysis of the correlation between CLSPN expression and DFI. A red line represents high CLSPN expression, and the blue lines represent the low CLSPN expression.

**Additional file 6: Figure S5.** Association between the CLSPN expression and progression-free interval (PFI) in cancer patients. (A) A forest plot of PFI association with CLSPN expression in 33 tumor types. (B-I) Kaplan–Meier survival curves of the association between CLSPN expression and PFI. A red line represents high CLSPN expression, and the blue lines represent the low CLSPN expression.

**Additional file 7: Figure S6.** The impact of single CLSPN CpG on LUAD prognosis. (A) The scatter plots of correlation between CLSPN expression and CLSPN methylation in different cancer types. (B) Kaplan—Meier plot for OS in LUAD patients with CLSPN-body-Island-cg00463507, CLSPN − TSS200 − Island − cg04263115, CLSPN − TSS1500 − Island − cg10246273 and CLSPN − 5'UTR;1stExon − Island − cg25109252 methylation. (C) Heatmap of CLSPN CpG methylation levels in LUAD by MethSurv. Rows represented the CpGs and columns represented the patients.

**Additional file 8: Figure S7.** Association between the CLSPN methylation and the OS of cancer patients from TCGA. (A) Kaplan–Meier survival curves of the correlation between the CLSPN methylation and OS in patients with BRCA, CESC, ESCA, GBM, HNSC, KIRC, SKCM and THCA. (B) Kaplan–Meier analysis of the correlation between CLSPN methylation and OS in patients with KICH, LIHC, READ, UVM and SARC.

**Additional file 9: Figure S8.** Correlation of CLSPN expression with stemness score in pan-cancer. (A) Radar map of the correlation between stemness and CLSPN expression. (B) The heatmap of correlation between mRNAsi/mDNAsi and CLSPN expression. (C) The scatter plots of association between CLSPN expression and mRNAsi/mDNAsi in different cancer types. *P < 0.05, **P < 0.01, ***P < 0.001.

**Additional file 10: Figure S9.** The distribution of CLSPN in LUAD at single-cell level using TISCH database. (A). Heatmap displayed the distribution of CLSPN expression in different cell types from several databases. (B). Single-cell cluster map of CLSPN in NSCLC_EMTAB6149 dataset. (C) Single-cell cluster map of CLSPN in NSCLC_GSE127465 dataset. (D) Single-cell cluster map of CLSPN in NSCLC_GSE143423 dataset.

**Additional file 11: Figure S10.** The correlation between CLSPN expression and IC50 values of anti-cancer drugs based on CellMiner™ database.

## Data Availability

All datasets presented in this study are included in the article/Supplementary Material.

## Abbreviations

CCK-8: Cell Counting Kit-8
EDU: 5‐Ethynyl‐2’‐Deoxyuridine
TMB: Tumor mutational burden
MSI: Microsatellite instability
MMR: Mismatch repair
CDK: Cyclin-dependent kinase
LUAD: Lung adenocarcinoma
OS: Overall survival
DFS: Disease-free survival
DSS: Disease-specific survival
DFI: Disease-free interval
PFI: Progression-free interval
GSEA: Gene set enrichment analysis
IF: Immunofluorescence
FBS: Fetal bovine serum
RT-qPCR: RNA isolation and quantitative reverse transcription PCR
PVDF: Polyvinylidene fluoride
ANOVA: One-way analysis of variance
GEO: Gene Expression Omnibus
ICB: Immune checkpoint blockade
ACT: Adoptive cell therapy
TME: Tumor Microenvironment
