# Supplementary figures and images for: A multi-omics analysis reveals CLSPN is associated with prognosis, immune microenvironment and drug resistance in cancers
Source: Biol Proced Online. 2023 Jun 3;25:16. doi: 10.1186/s12575-023-00201-6 (PMC10239117; doi:10.1186/s12575-023-00201-6)

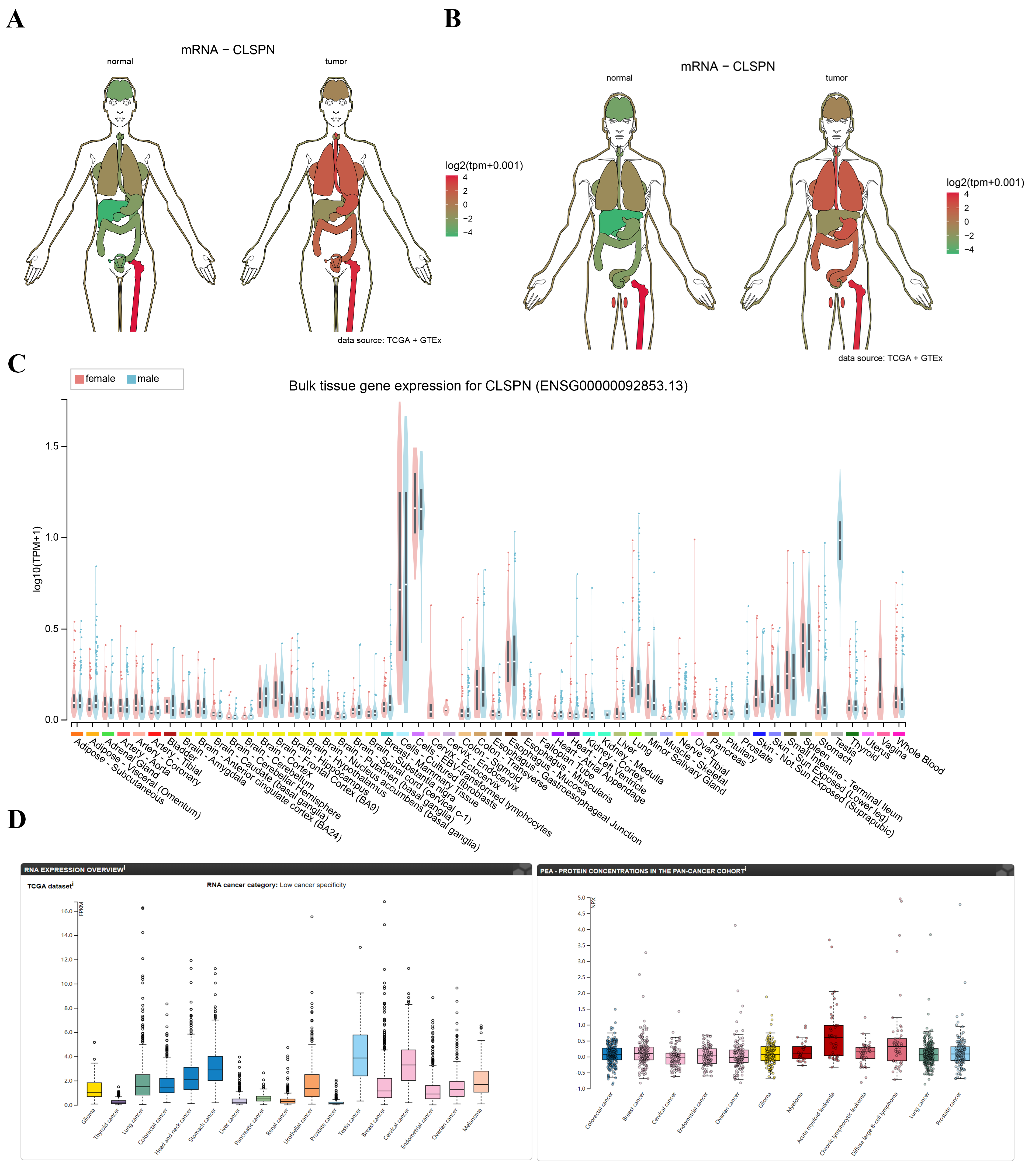

Supplement: Supplementary file 2 — Additional file 2: Figure S1. CLSPN expression in different tissues. (A & B) The CLSPN enrichments of different tissues in males and females were displayed by TCGA and GTEx database. (C) The mRNA expression levels of CLSPN in different genders. (D) The mRNA and protein expression of CLSPN in cancers downloaded from Human Protein Atlas. [file 12575_2023_201_MOESM2_ESM.tif]

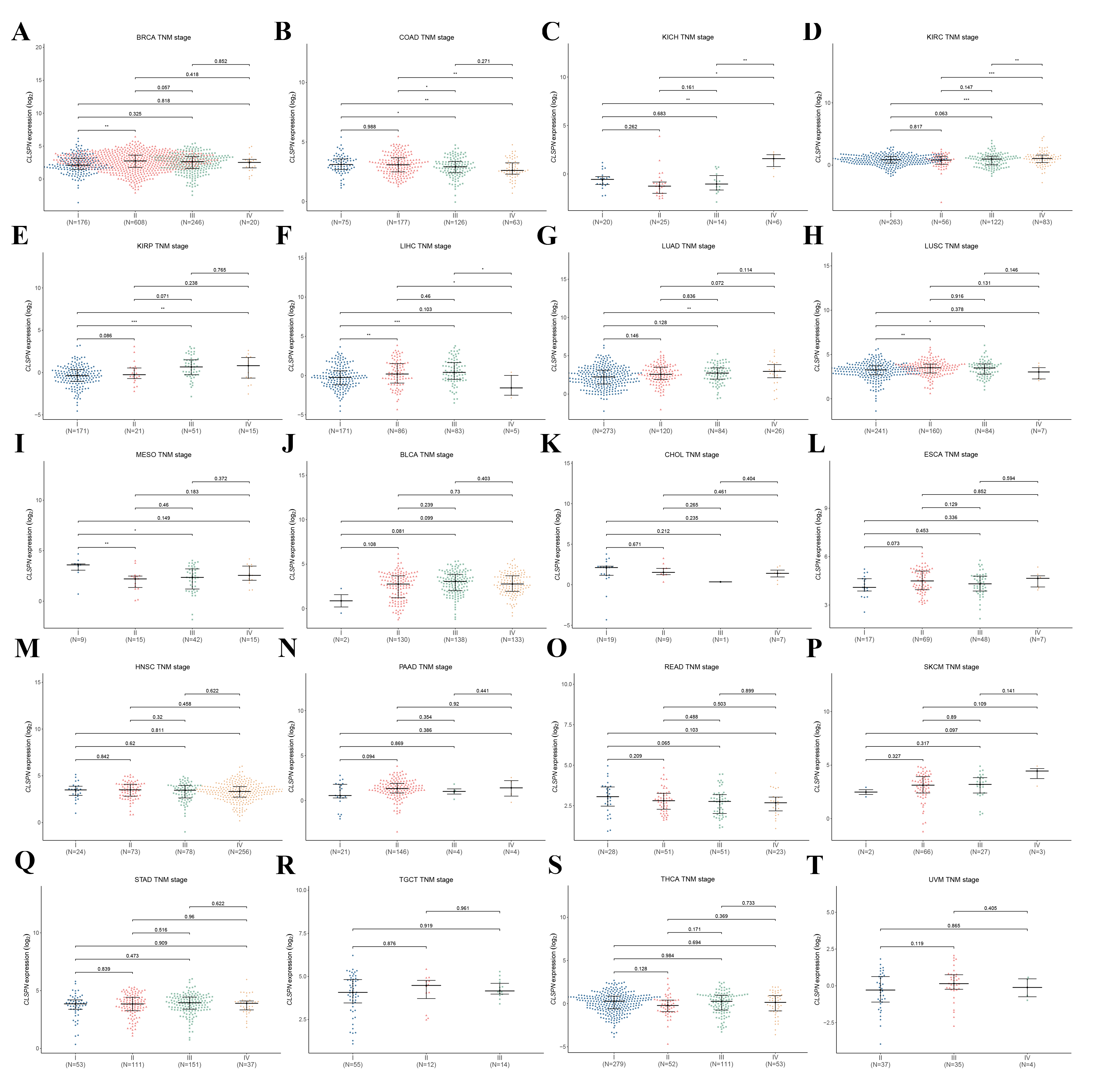

Supplement: Supplementary file 3 — Additional file 3: Figure S2. Association between CLSPN expression and tumor stage in (A) BRCA; (B) COAD; (C) KICH; (D) KIRC; (E) KIRP; (F) LIHC; (G) LUAD; (H) LUSC; (I) MESO;(J) BLCA; (K) CHOL; (L) ESCA; (M) HNSC; (N) PAAD; (O) READ; (P) SKCM; (Q) STAD; (R) TGCT; (S) THCA; (T) UVM. *P < 0.05, **P < 0.01, and ***P < 0.001. [file 12575_2023_201_MOESM3_ESM.tif]

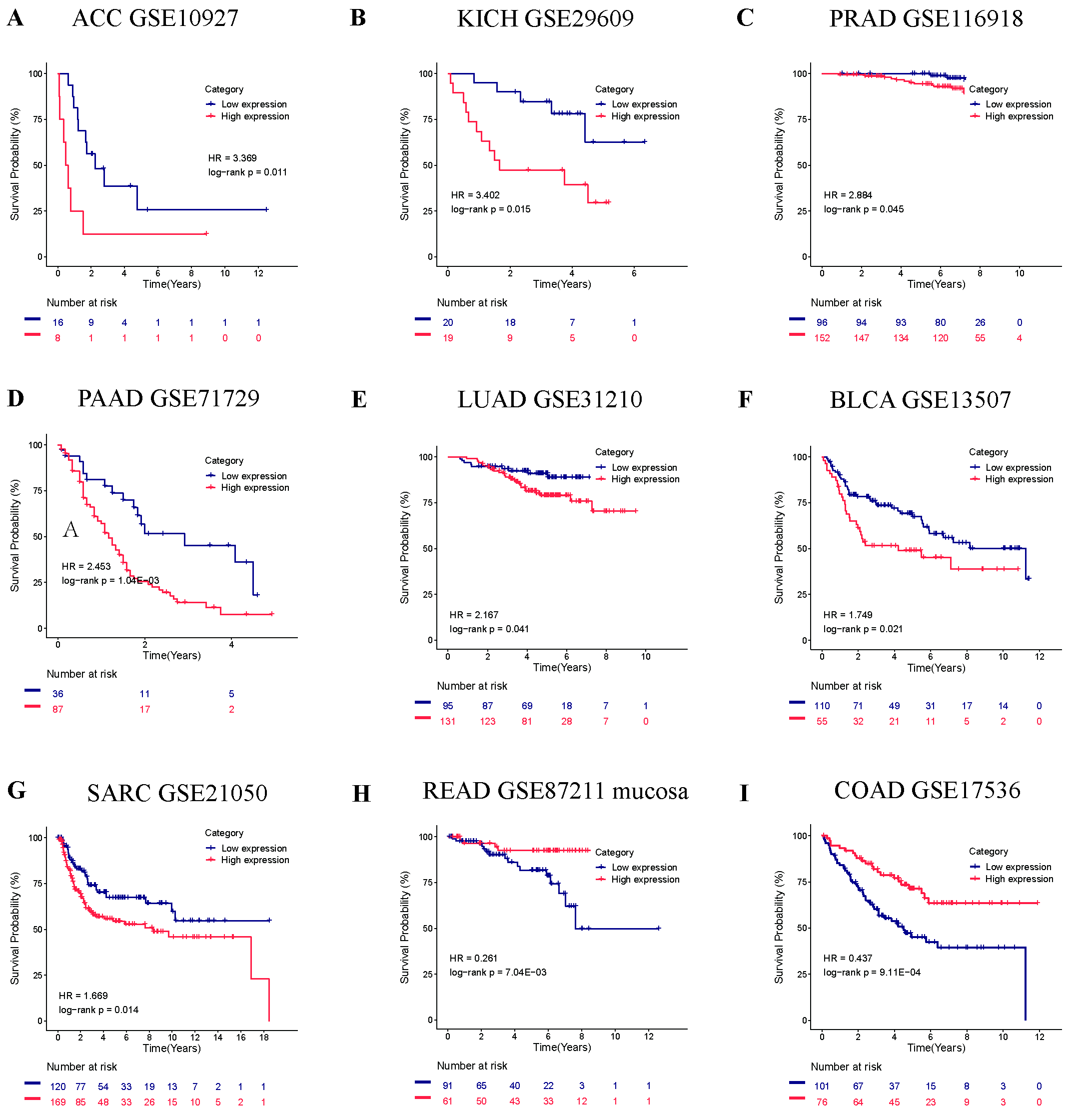

Supplement: Supplementary file 4 — Additional file 4: Figure S3. Kaplan–Meier analysis of the correlation between CLSPN expression and the OS of cancer patients with (A) ACC, (B) KICH, (C) PRAD, (D) PAAD, (E) LUAD, (F) BLCA, (G) SARC, (H) READ and (I) COAD in GEO clinical cohort. A red line represents high CLSPN expression, and the blue lines represent the low CLSPN expression. [file 12575_2023_201_MOESM4_ESM.tif]

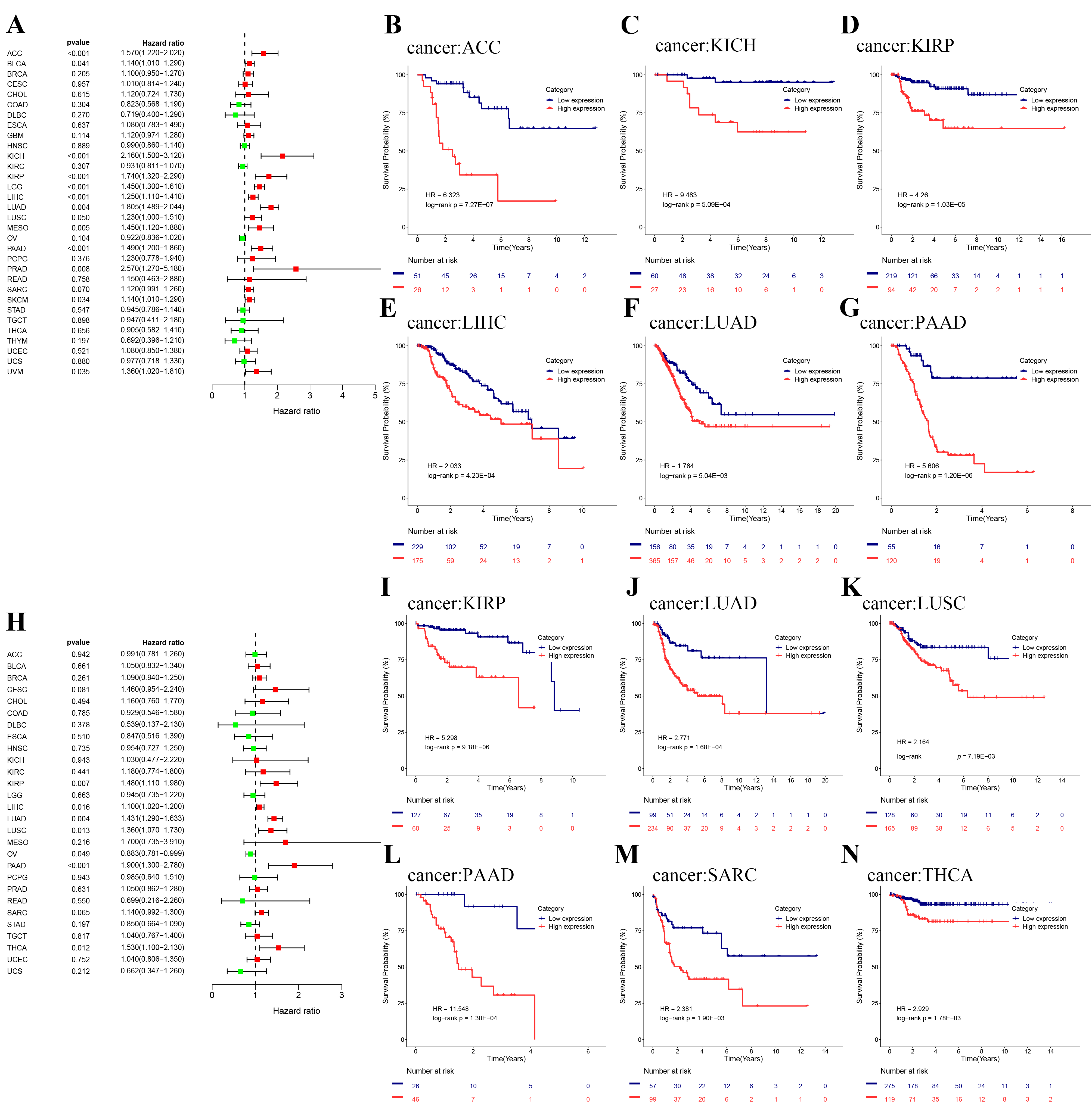

Supplement: Supplementary file 5 — Additional file 5: Figure S4. Association between CLSPN expression levels and disease-specific survival (DSS) and disease-free interval (DFI) of cancer patients. (A) Forest plot of the association of CLSPN expression and DSS in 33 types of tumor. (B–G) Kaplan–Meier analysis of the association between CLSPN expression and DSS. (H) Forest plot of the association of CLSPN expression and DFI in 33 types of tumor. (I–N) Kaplan–Meier analysis of the correlation between CLSPN expression and DFI. A red line represents high CLSPN expression, and the blue lines represent the low CLSPN expression. [file 12575_2023_201_MOESM5_ESM.tif]

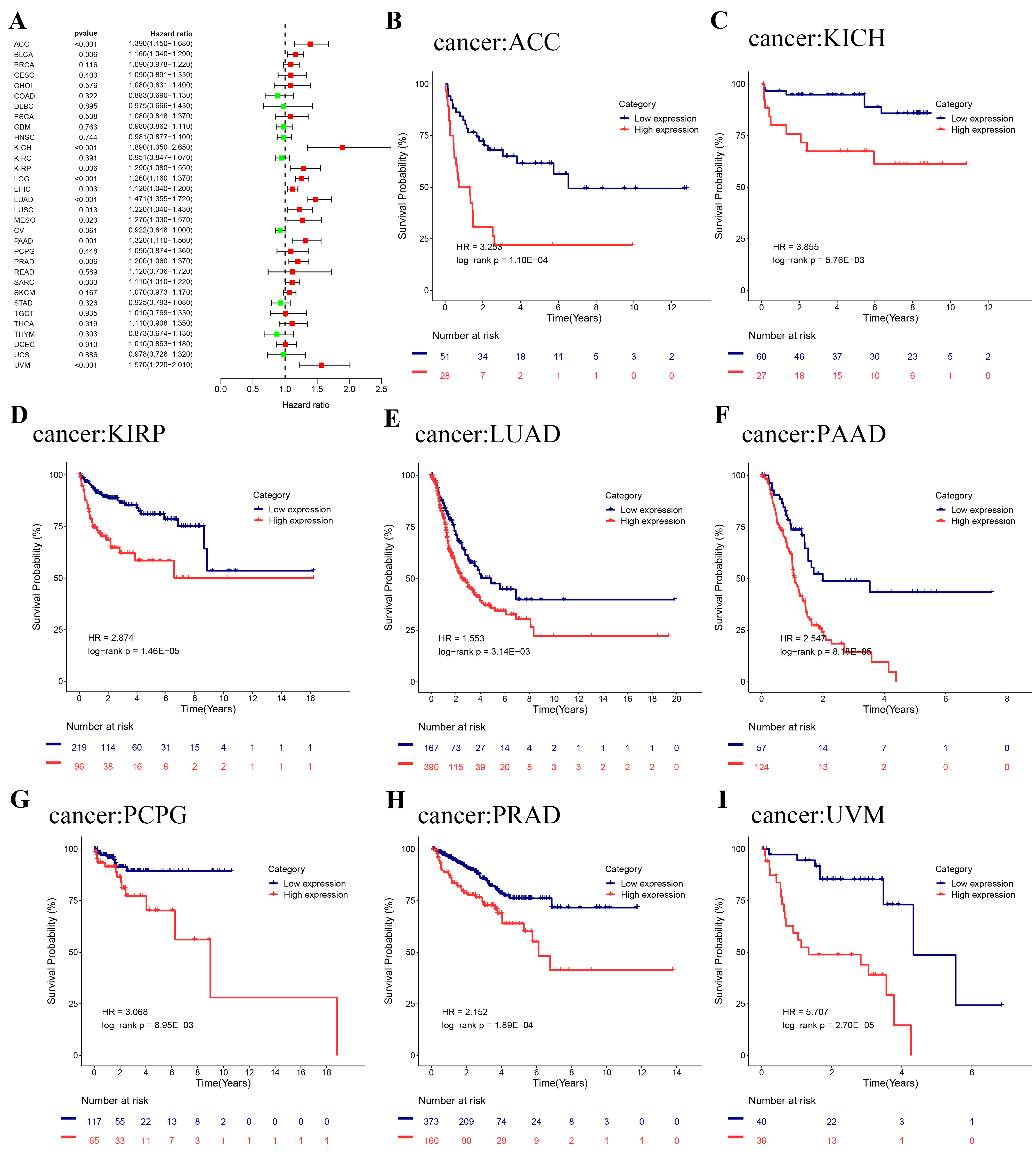

Supplement: Supplementary file 6 — Additional file 6: Figure S5. Association between the CLSPN expression and progression-free interval (PFI) in cancer patients. (A) A forest plot of PFI association with CLSPN expression in 33 tumor types. (B-I) Kaplan–Meier survival curves of the association between CLSPN expression and PFI. A red line represents high CLSPN expression, and the blue lines represent the low CLSPN expression. [file 12575_2023_201_MOESM6_ESM.tif]

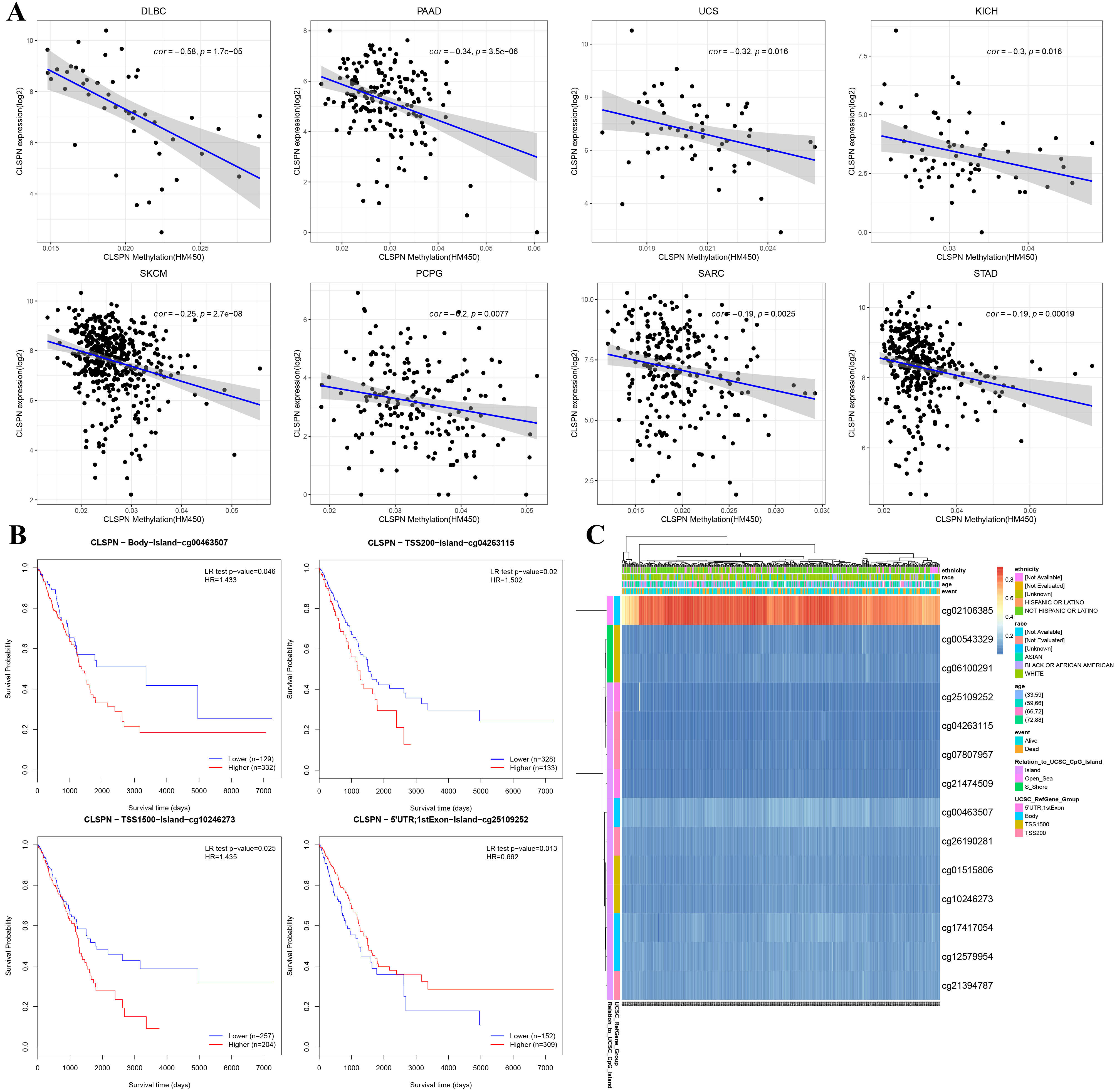

Supplement: Supplementary file 7 — Additional file 7: Figure S6. The impact of single CLSPN CpG on LUAD prognosis. (A) The scatter plots of correlation between CLSPN expression and CLSPN methylation in different cancer types. (B) Kaplan—Meier plot for OS in LUAD patients with CLSPN-body-Island-cg00463507, CLSPN − TSS200 − Island − cg04263115, CLSPN − TSS1500 − Island − cg10246273 and CLSPN − 5'UTR;1stExon − Island − cg25109252 methylation. (C) Heatmap of CLSPN CpG methylation levels in LUAD by MethSurv. Rows represented the CpGs and columns represented the patients. [file 12575_2023_201_MOESM7_ESM.tif]

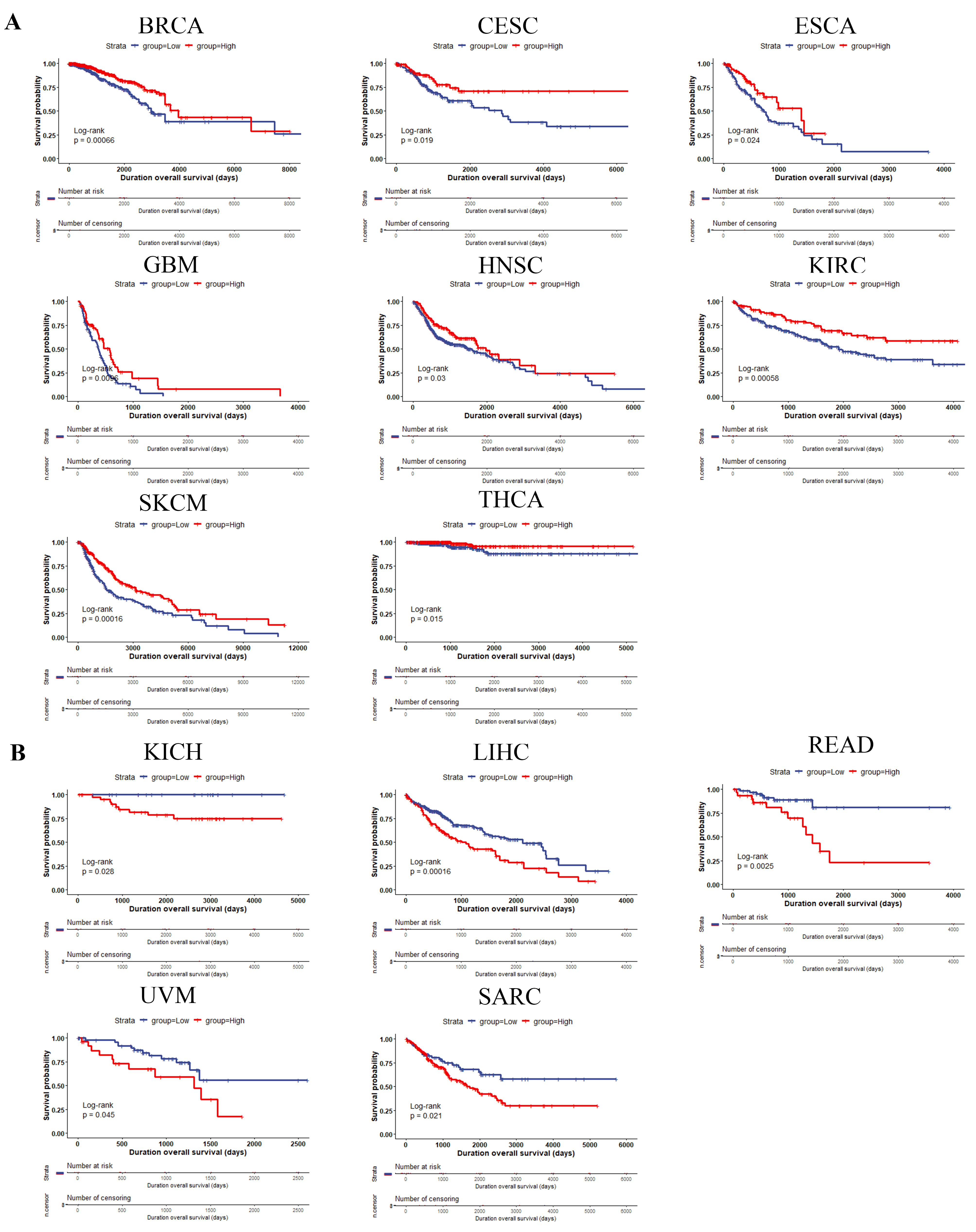

Supplement: Supplementary file 8 — Additional file 8: Figure S7. Association between the CLSPN methylation and the OS of cancer patients from TCGA. (A) Kaplan–Meier survival curves of the correlation between the CLSPN methylation and OS in patients with BRCA, CESC, ESCA, GBM, HNSC, KIRC, SKCM and THCA. (B) Kaplan–Meier analysis of the correlation between CLSPN methylation and OS in patients with KICH, LIHC, READ, UVM and SARC. [file 12575_2023_201_MOESM8_ESM.tif]

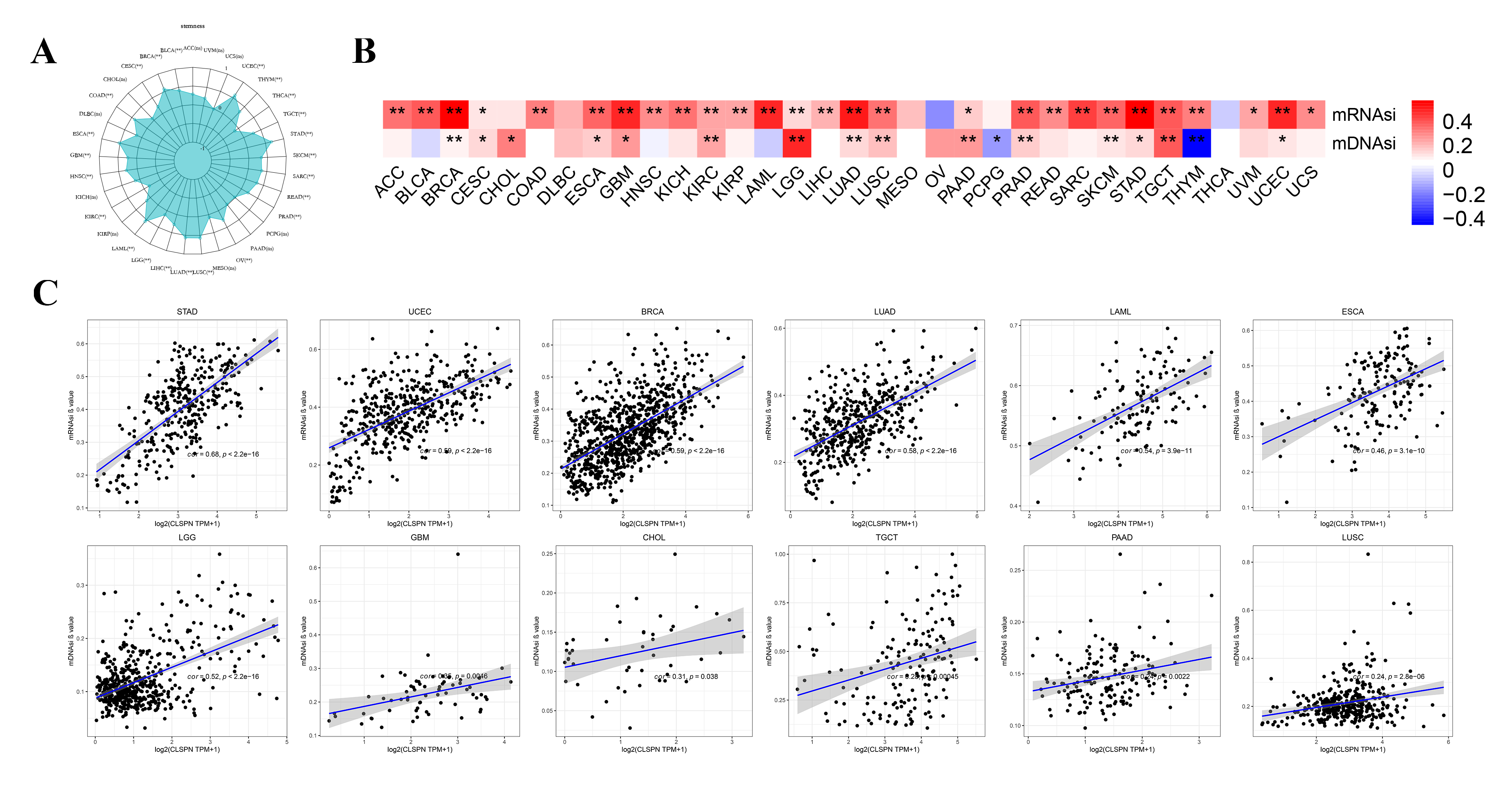

Supplement: Supplementary file 9 — Additional file 9: Figure S8. Correlation of CLSPN expression with stemness score in pan-cancer. (A) Radar map of the correlation between stemness and CLSPN expression. (B) The heatmap of correlation between mRNAsi/mDNAsi and CLSPN expression. (C) The scatter plots of association between CLSPN expression and mRNAsi/mDNAsi in different cancer types. *P < 0.05, **P < 0.01, ***P < 0.001. [file 12575_2023_201_MOESM9_ESM.tif]

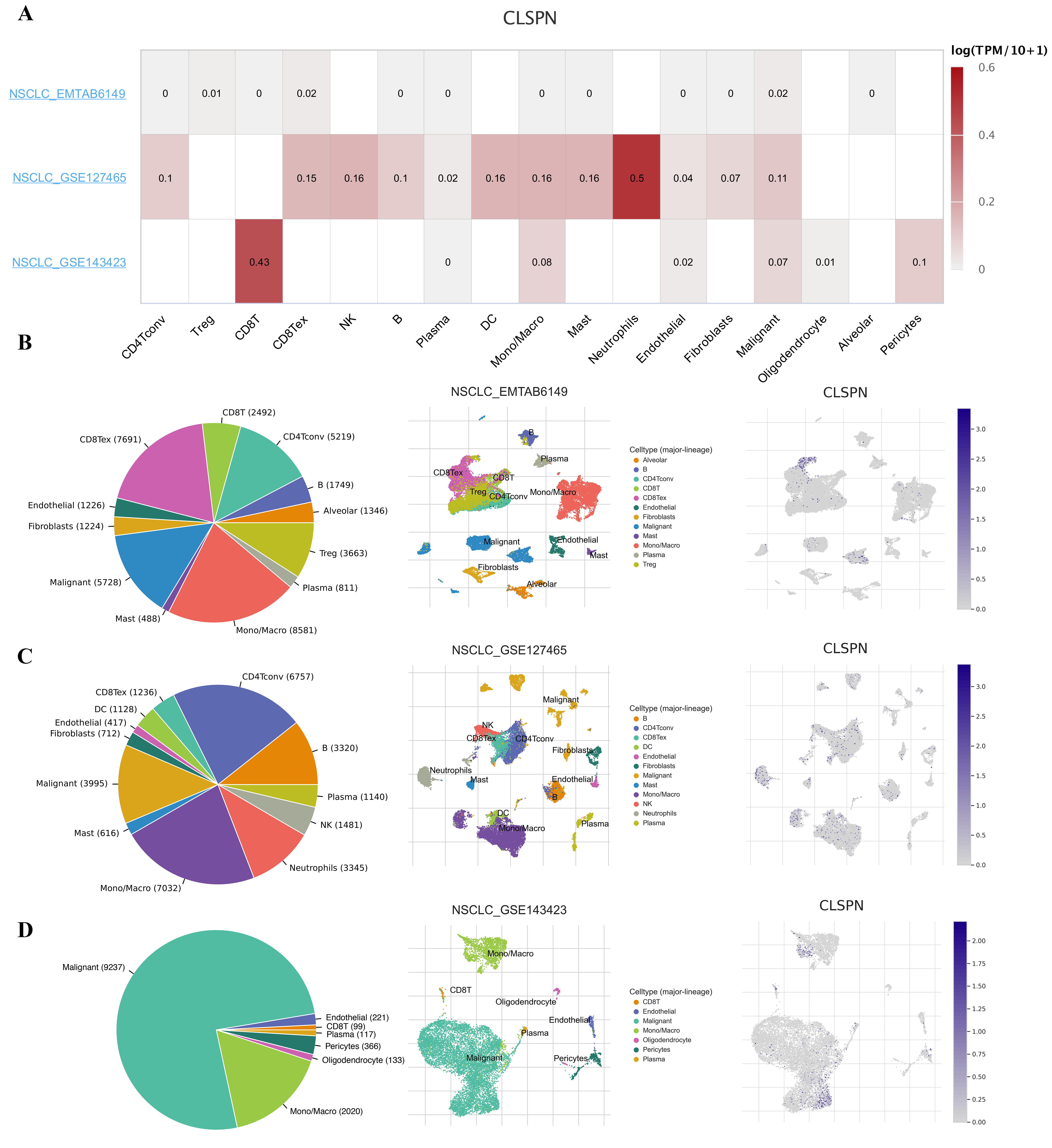

Supplement: Supplementary file 10 — Additional file 10: Figure S9. The distribution of CLSPN in LUAD at single-cell level using TISCH database. (A). Heatmap displayed the distribution of CLSPN expression in different cell types from several databases. (B). Single-cell cluster map of CLSPN in NSCLC_EMTAB6149 dataset. (C) Single-cell cluster map of CLSPN in NSCLC_GSE127465 dataset. (D) Single-cell cluster map of CLSPN in NSCLC_GSE143423 dataset. [file 12575_2023_201_MOESM10_ESM.tif]

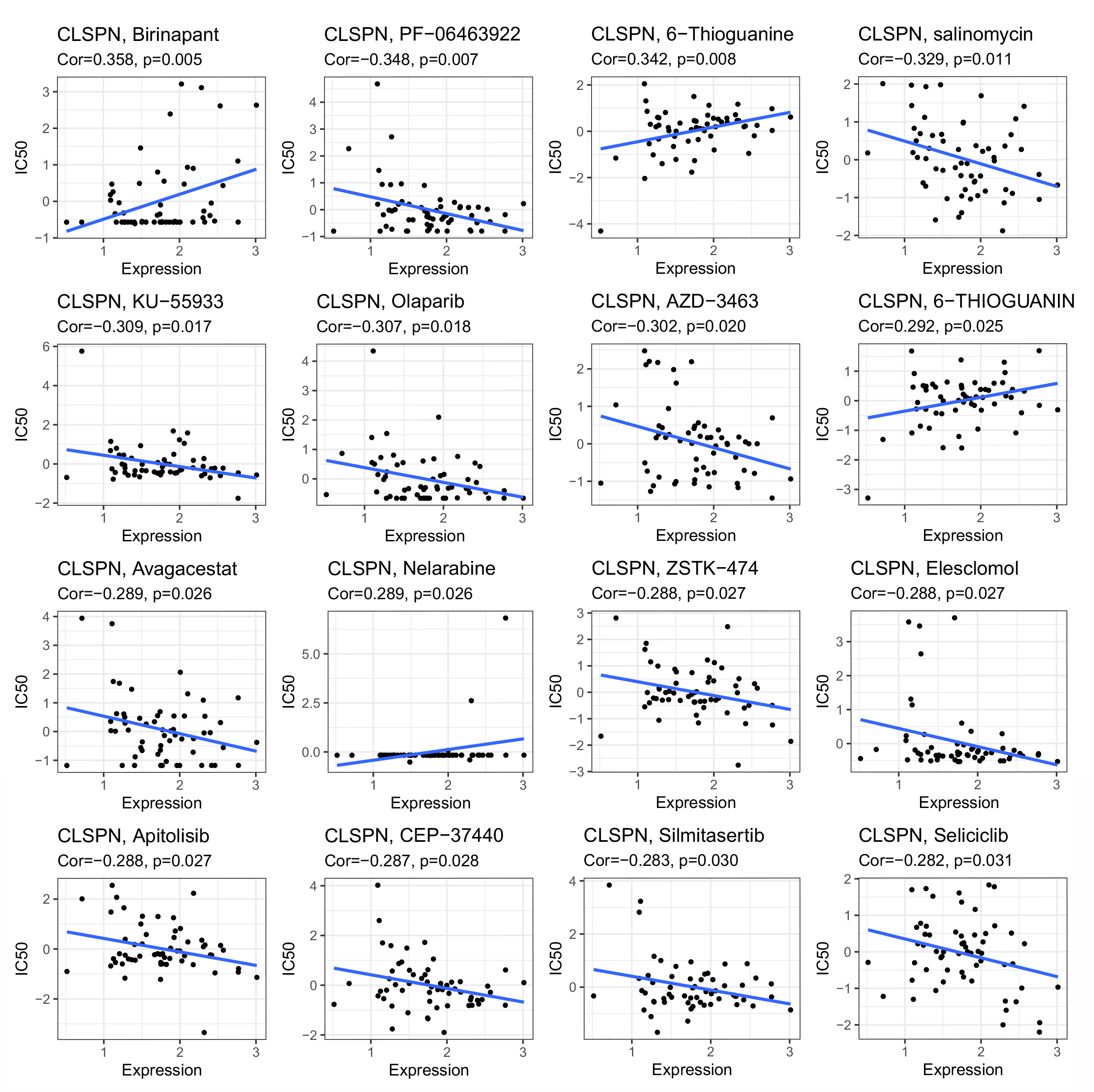

Supplement: Supplementary file 11 — Additional file 11: Figure S10. The correlation between CLSPN expression and IC50 values of anti-cancer drugs based on CellMiner™ database. [file 12575_2023_201_MOESM11_ESM.tif]
